# *ECH1*-Deficiency-Mediated Polyunsaturated Fatty Acid Accumulation Promotes Vascular Smooth Muscle Cell Ferroptosis and Chronic-Kidney-Disease-Associated Plaque Vulnerability

**DOI:** 10.34133/research.1411

**Published:** 2026-09-02

**Authors:** Xianjin Bi, Jinrun Zhou, Liang Liu, Zijin Chen, Bo Liang, Shihua Tian, Jiachuan Xiong, Qigang Lan, Liangjing Lv, Yan Li, Tangli Xiao, Ting He, Jun Zhang, Ling Nie, Jinghong Zhao

**Affiliations:** ^1^ Department of Nephrology, the Key Laboratory for the Prevention and Treatment of Chronic Kidney Disease of Chongqing, Chongqing Clinical Research Center of Kidney and Urology Diseases, Xinqiao Hospital, Army Medical University (Third Military Medical University), Chongqing 400037, China.; ^2^ Key Laboratory of Geriatric Cardiovascular and Cerebrovascular Disease Research (Army Medical University), Ministry of Education, Chongqing 400037, China.; ^3^ Department of Cardio-Renal Department, People’s Liberation Army Joint Logistic Support Force 945th Hospital, Ya’an 625000, China.

## Abstract

Vulnerable atherosclerotic plaques represent a critical pathological basis of acute cardiocerebrovascular events. Previous studies indicate that atherosclerotic plaques are more vulnerable under chronic kidney disease (CKD) milieus. Emerging evidence highlights that vascular smooth muscle cells (VSMCs) play a pivotal role in maintaining plaque stability, but the underlying mechanisms remain incompletely elucidated. Here, single-cell sequencing and functional enrichment analysis of arterial tissues from patients with CKD are performed, identifying lipid metabolism disorders in VSMCs. Further utilizing spatial and targeted lipidomics, apolipoprotein-E-deficient (*ApoE*^−/−^) mouse with CKD (CKD/*ApoE*^−/−^ mouse) and bioinformatics analysis, we investigate the lipidomic profile of VSMCs and find that VSMCs in CKD-associated vulnerable plaques exhibit significant accumulation of polyunsaturated fatty acids (PUFAs), which induces VSMC ferroptosis and exacerbates plaque vulnerability. Mechanistically, the deficiency of *ECH1* leads to the accumulation of PUFA in VSMCs, thereby inducing ferroptosis in fibrous cap VSMCs and fibrous cap thinning. Meanwhile, low expression of mRNA binding protein human antigen R (HuR) resulting from CKD milieus mediates the lack of *ECH1* in VSMCs. In contrast, VSMC-specific overexpression of *ECH1*, inhibition of PUFA release using giripladib or suppression of PUFA lipid peroxidation with PRGL493 significantly alleviate VSMC ferroptosis and CKD-associated plaque vulnerability. These findings reveal that *ECH1*-deficiency-driven PUFA accumulation is responsible for VSMC ferroptosis and atherosclerotic plaque vulnerability. Targeted regulation of *ECH1*-mediated PUFA metabolism may be a promising preventive and therapeutic strategy for CKD-associated plaque vulnerability.

## Introduction

Cardiocerebrovascular diseases are the leading cause of mortality worldwide, accounting for approximately 19.8 million deaths each year and nearly one-third of all deaths globally [1,2]. Vulnerable atherosclerotic plaques can trigger the formation of thrombus and subsequent cardiovascular and cerebrovascular events [3]. Recent study indicates that vulnerable plaques account for over 50% of cases in atherosclerotic disease [4]. Therefore, elucidating the mechanisms of vulnerable atherosclerotic plaques is crucial for identifying intervention targets in cardiocerebrovascular diseases.

Vulnerable atherosclerotic plaques are primarily characterized by a thin fibrous cap, a large necrotic core, and significant inflammatory cell infiltration [5,6]. Previous study confirms that the fibrous cap, mainly composed of vascular smooth muscle cells (VSMCs) and VSMC-produced matrix, is a primary determinant of plaque stability [7]. Fibrous cap VSMCs are the subset of VSMCs that reside within the fibrous cap structure of atherosclerotic plaques. They are predominantly derived from contractile VSMCs in the tunica media: Following phenotypic dedifferentiation, these medial VSMCs migrate across the internal elastic lamina into the intima and undergo local proliferation to accumulate in the fibrous cap region. Fibrous cap VSMCs serve as a critical atheroprotective cellular population that maintains the mechanical tensile strength of the plaque and protects against plaque rupture and subsequent atherothrombotic events. These cells predominantly exhibit a synthetic phenotype while retaining the expression of a subset of canonical contractile markers, such as actin alpha 2, smooth muscle (ACTA2) and transgelin (TAGLN) [8,9]. Typically, a reduction in the number of fibrous cap VSMCs aggravates fibrous cap thinning, thereby promoting plaque vulnerability and rupture [10]. Compared with patients without chronic kidney disease (CKD), patients with CKD exhibit more severe fibrous cap thinning. Our prior research demonstrates that VSMC senescence plays a pivotal role in the pathogenesis of plaque vulnerability [11]. However, inhibition of VSMC senescence only partially ameliorates atherosclerotic plaque vulnerability [11], indicating the involvement of additional mechanisms.

Lipid metabolism disorder is primarily related to progression of atherosclerosis and plaque stability [12,13]. Previous research has extensively investigated the role of VSMCs in lipid metabolism disorder and their contribution to atherosclerosis, with a particular focus on the mechanisms of formation of lipid-loaded VSMCs [14]. This process involves 4 core biological steps: the uptake of atherogenic lipoproteins by VSMCs, the intracellular esterification of cholesterol, hydrolysis of cholesteryl esters, and the efflux of cholesterol [15]. Based on this pathological mechanism, current treatment strategies primarily use statins and proprotein convertase subtilisin/kexin type 9 (PCSK9) inhibitors to reduce the amount of lipoprotein that can infiltrate the vessel wall and be engulfed by VSMCs [16,17]. However, despite intensive lipid-lowering therapy, the incidence of thrombotic events remains markedly high, often referred to as residual risk [18]. This clinical challenge indicates a limitation in current therapies that focus mainly on regulating the quantity of lipids. Therefore, precise characterization of the lipid profile of VSMCs within atherosclerotic plaques is essential for elucidating the mechanism of plaque vulnerability [14]. Nevertheless, the precise mechanisms driving lipid metabolism abnormalities in VSMCs and their role in plaque vulnerability remain incompletely understood.

In this study, we observed that fibrous cap VSMCs in CKD/*ApoE*^−/−^ mice displayed an accumulation of polyunsaturated fatty acids (PUFAs), which consequently induced VSMC ferroptosis and fibrous cap thinning. The deficiency of enoyl-coenzyme A hydratase 1 (*ECH1*) was confirmed as the prominent cause of PUFA accumulation in fibrous cap VSMCs. Regulating *ECH1*-mediated PUFA metabolism might be a critical avenue to inhibit VSMC ferroptosis and CKD-associated plaque vulnerability.

## Results

### PUFA accumulation is observed in fibrous cap VSMCs of CKD/*ApoE*^−/−^ mice

**Fig. 1.**
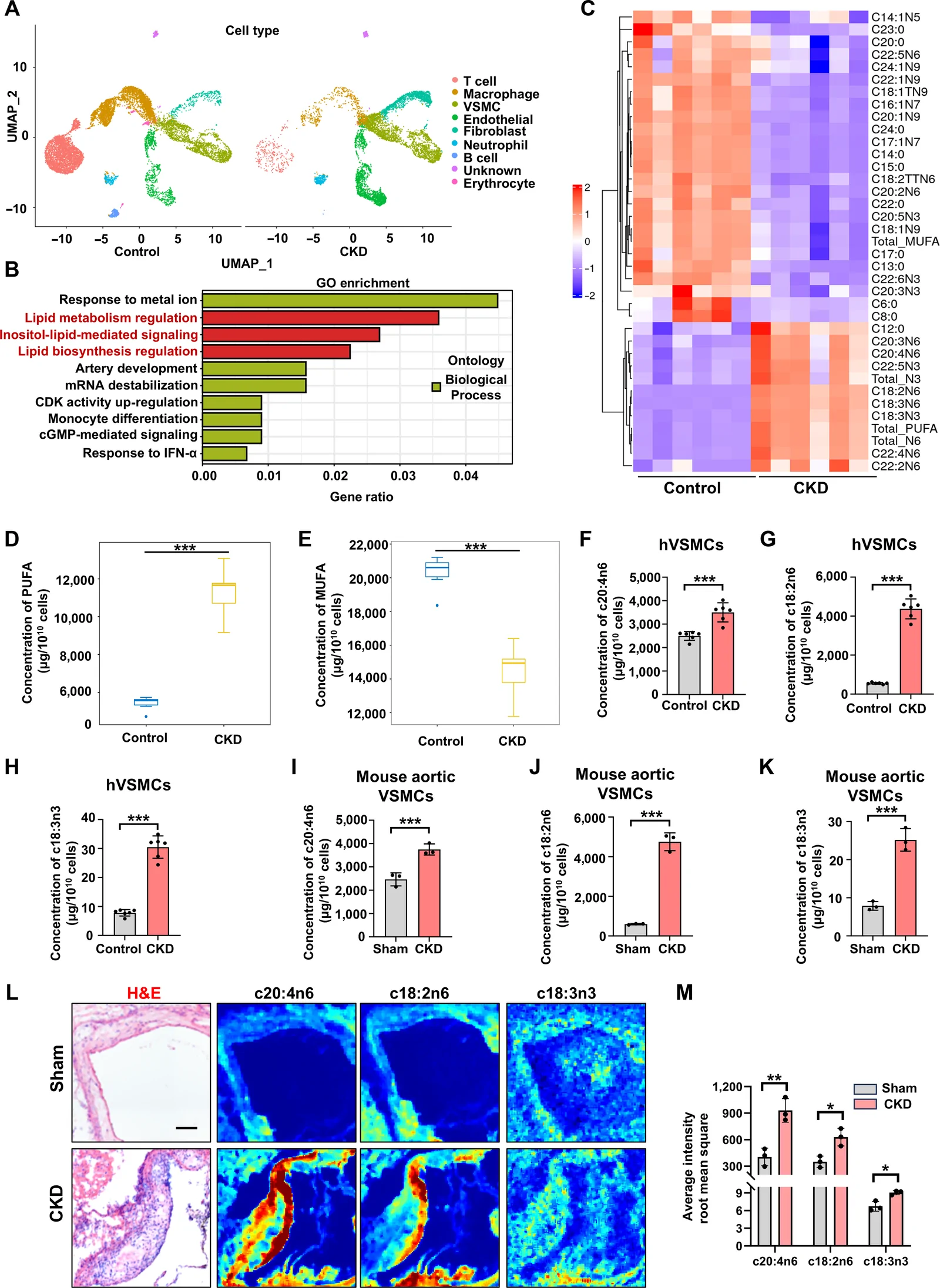
Polyunsaturated fatty acid (PUFA) accumulation is observed in fibrous cap vascular smooth muscle cells (VSMCs) of chronic kidney disease (CKD)/*ApoE*^−/−^ mice. (A) Different cell types and clusters were identified in arterial tissues from control and patients with CKD through single-cell RNA sequencing. (B) Gene Ontology (GO) functional enrichment analysis of differentially expressed genes (DEGs) in VSMCs of single-cell RNA sequencing. cGMP, cyclic guanosine monophosphate; IFN-α, interferon-α. (C) Heatmap of targeted lipidomics analysis in human VSMCs (hVSMCs) after incubation with normal or CKD serum. (D and E) Quantification of the level of PUFA or monounsaturated fatty acid (MUFA) in hVSMCs after incubation with normal or CKD serum (*n* = 6). (F to H) Quantification of the level of arachidonic acid (AA), linoleic acid (LA), and linolenic acid (ALA) in hVSMCs after incubation with normal or CKD serum (*n* = 6). (I to K) Quantification of the level of AA, LA, and ALA in aortic VSMCs of sham/*ApoE*^−/−^ and CKD/*ApoE*^−/−^ mice (*n* = 6). (L) Representative hematoxylin and eosin (H&E) staining and spatial targeted lipidomic image in AA, LA, and ALA of the fraction area of aortic root plaques in sham/*ApoE*^−/−^ and CKD/*ApoE*^−/−^ mice. Scale bars, 100 μm. (M) Quantification of average intensity of root mean square of spatial targeted lipidomic image in AA, LA, and ALA (*n* = 3). Data represent means ± SD. *P < 0.05, **P < 0.01, and ***P < 0.001, 2-tailed Student’s t test.

To investigate the potential mechanism of VSMCs in CKD-associated plaque vulnerability, we conducted single-cell sequencing on arterial vascular tissues from patients with CKD (Fig. 1A). Gene Ontology (GO) functional enrichment analysis identified lipid metabolism disorders in VSMCs (Fig. 1B). The expression distributions of well-established lineage-specific marker genes across all annotated cell populations are presented as violin plots, verifying the accuracy of cell type annotation for human vascular single-cell RNA sequencing data (Fig. S1A). To address the lipidomic profile of VSMCs in CKD-associated plaque vulnerability, we performed the targeted lipidomics analysis, revealing a significant accumulation of PUFA and an obvious decrease in monounsaturated fatty acid (MUFA) in CKD-serum-cultured human VSMCs (hVSMCs) (Fig. 1C to E). Specifically, the levels of ferroptosis-associated PUFAs [19,20], including linoleic acid (LA; c18:2n6), linolenic acid (ALA; c18:3n3), and arachidonic acid (AA; c20:4n6), were significantly elevated (Fig. 1F to H) in hVSMCs. Consistently, the levels of AA, LA, and ALA were markedly increased in arterial VSMCs isolated from CKD/*ApoE*^−/−^ mice (Fig. 1I to K) and in mouse VSMCs treated with serum from CKD/*ApoE*^−/−^ mice (Fig. S1B to D). Spatial targeted lipidomic detection further confirmed the accumulation of PUFA in fibrous cap VSMCs in CKD/*ApoE*^−/−^ mice (Fig. 1L and M). These results indicated that the lipid profile of fibrous cap VSMCs in CKD-associated vulnerable plaques is characterized by the accumulation of PUFA.

### PUFA accumulation promotes ferroptosis in fibrous cap VSMCs of CKD/*ApoE*^−/−^ mice

**Fig. 2.**
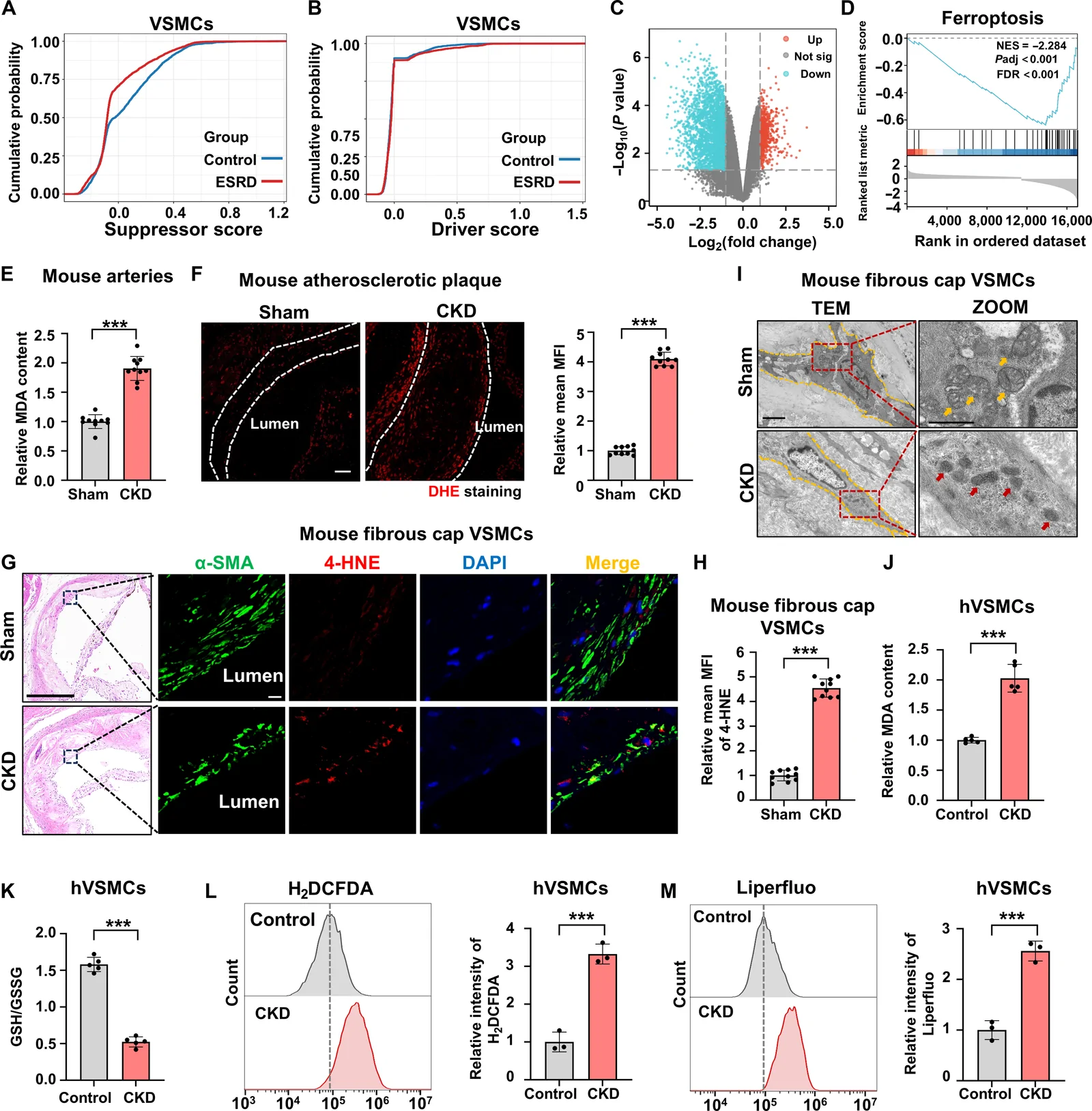
Polyunsaturated fatty acid (PUFA) accumulation promotes ferroptosis in fibrous cap vascular smooth muscle cells (VSMCs) of chronic kidney disease (CKD)/*ApoE*^−/−^ mice. (A and B) Cumulative distribution function analysis of ferroptosis suppressor score of VSMCs in control and patients with CKD. (C) The volcano plot shows the differentially expressed genes in aortic VSMCs between sham/*ApoE*^−/−^ and CKD/*ApoE*^-/−^ mice. (D) Gene set enrichment analysis (GSEA) analysis of ferroptosis gene set in aortic VSMCs of Sham/*ApoE*^−/−^ and CKD/*ApoE*^−/−^ mice. NES, normalized enrichment score; FDR, false discovery rate. (E) Relative of malondialdehyde (MDA) content in arteries of sham/*ApoE*^−/−^ and CKD/*ApoE*^−/−^ mice (*n* = 10). (F) Representative images and quantification of relative mean fluorescence intensity (MFI) of dihydroethidium (DHE) staining in aortic root plaques of sham/*ApoE*^−/−^ and CKD/*ApoE*^−/−^ mice (*n* = 10). Scale bar, 100 μm. (G) Representative images of hematoxylin and eosin (H&E), 4-hydroxynonenal (4-HNE), and α-smooth muscle actin (α-SMA) staining in fibrous cap VSMCs of sham/*ApoE*^−/−^ and CKD/*ApoE*^−/−^ mice. Scale bars, 200 μm (H&E) and 10 μm (immunofluorescence [IF]). (H) Quantification of relative mean MFI of 4-HNE in fibrous cap VSMCs of sham/*ApoE*^−/−^ and CKD/*ApoE*^−/−^ mice (*n* = 10). (I) Representative transmission electron microscopy (TEM) images of fibrous cap VSMCs in sham/*ApoE*^−/−^ and CKD/*ApoE*^−/−^ mice. Scale bar, 2 μm. (J and K) Relative of MDA content and the ratio of reduced glutathione (GSH)/oxidized glutathione (GSSG) in human VSMCs (hVSMCs) after incubation with normal or CKD serum (*n* = 5). (L and M) Representative flow cytometry analysis and the MFI of reactive oxygen species (ROS) and Liperfluo in hVSMCs incubated with normal or CKD serum (*n* = 3). Data represent means ± SD. ***P < 0.001, 2-tailed Student’s *t* test.

Based on single-cell sequencing analysis, the cumulative distribution function revealed a significant reduction in the ferroptosis suppressor score of VSMCs in patients with end-stage renal disease (ESRD), which is consistent with the accumulation of PUFA triggering ferroptosis (Fig. 2A and B). Accordingly, gene set enrichment analysis (GSEA) of our published single-VSMC microarray data (GSE135626) identified significant enrichment of ferroptosis-associated pathways in VSMCs under CKD milieus (Fig. 2C and D). In CKD/*ApoE*^−/−^ mice, accelerated atherosclerosis, fibrous cap thinning, reduced VSMC number, decreased intraplaque collagen content, and enlarged necrotic core area were observed, accompanied by significantly shortened vessel occlusion time in the ferric chloride (FeCl_3_)-induced carotid thrombosis assay (Fig. S2A to E). We observed marked elevations in both malondialdehyde (MDA) (Fig. 2E) and reactive oxygen species (ROS) levels (Fig. 2F) in arteries and fibrous cap of CKD/*ApoE*^−/−^ mice. Meanwhile, 4-hydroxynonenal (4-HNE) expression was significantly increased in fibrous cap VSMCs of CKD/*ApoE*^−/−^ mice (Fig. 2G and H), accompanied by ferroptosis-associated morphological changes, including mitochondrial shrinkage, increased membrane density, and reduced or absent cristae (Fig. 2I). Moreover, the expression of glutathione peroxidase 4 (GPX4) and solute carrier family 7 member 11 (SLC7A11) were both significantly down-regulated in atherosclerotic vessels of CKD/*ApoE*^−/−^ mice (Fig. S2F to J). In vitro, CKD serum incubation induced ferroptosis-associated morphological alterations, the elevation of MDA, ROS, lipid ROS, and oxidized boron-dipyrromethene (BODIPY) fluorescence intensity, as well as decreased reduced glutathione (GSH)/oxidized glutathione (GSSG) ratio and down-regulated expression of GPX4 and SLC7A11 in hVSMCs and primary mouse VSMCs (Fig. 2J to M and Fig. S3A to L). In summary, these findings confirmed that PUFA accumulation in fibrous cap VSMCs under CKD milieus could induce lipid peroxidation and ferroptosis.

**Fig. 3.**
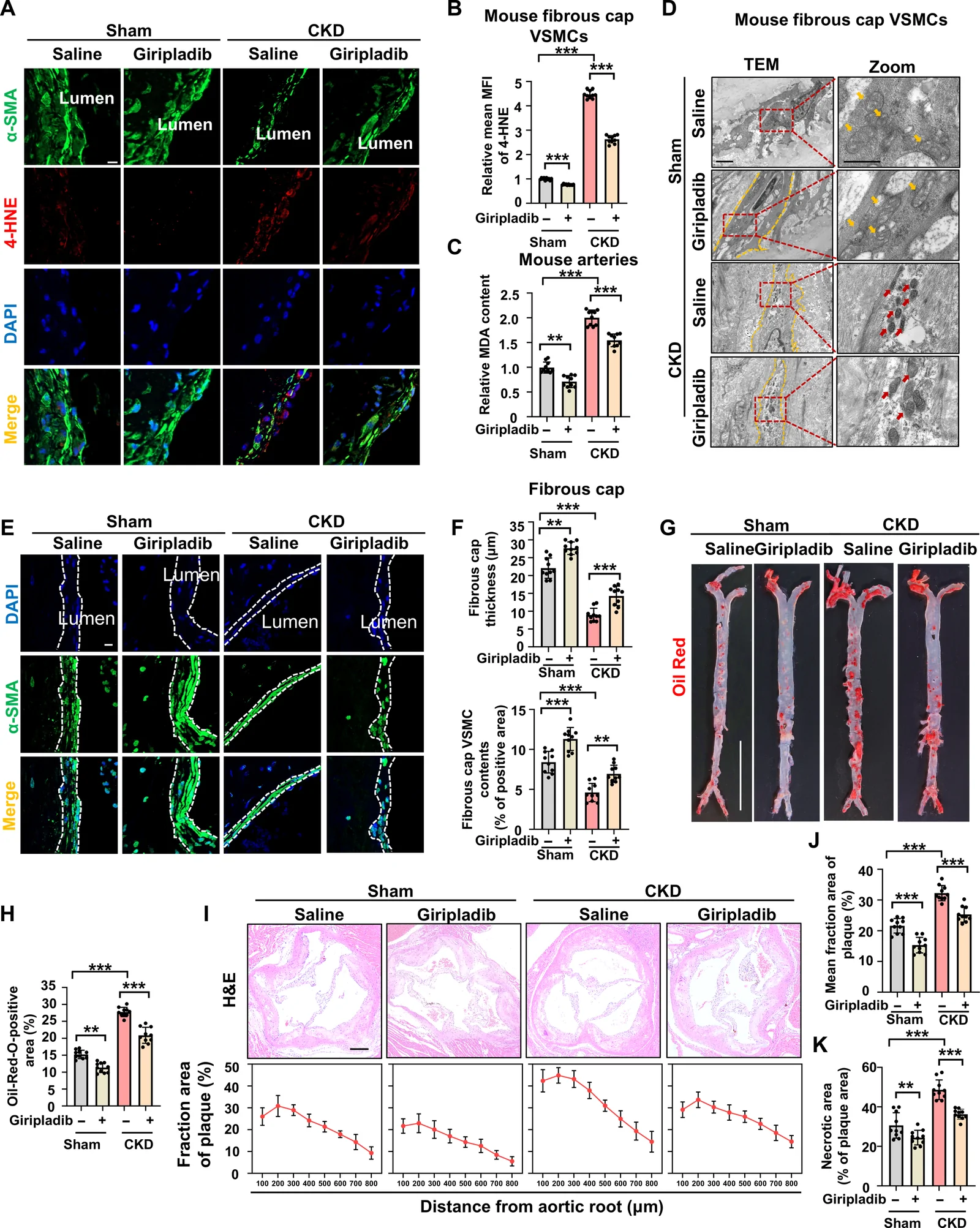
Pharmacological inhibition of cytosolic phospholipase A2 (cPLA2) mitigates vascular smooth muscle cell (VSMC) ferroptosis and plaque vulnerability. (A and B) Representative images and quantification of relative mean fluorescence intensity (MFI) of α-smooth muscle actin (α-SMA) and 4-hydroxynonenal (4-HNE) staining in fibrous cap VSMCs of sham/*ApoE*^−/−^ and chronic kidney disease (CKD)/*ApoE*^−/−^ mice after giripladib treatment (*n* = 10). Scale bar, 10 μm. (C) Relative of malondialdehyde (MDA) content in arteries of sham/*ApoE*^−/−^ and CKD/*ApoE*^−/−^ mice after giripladib treatment (*n* = 10). (D) Representative transmission electron microscopy (TEM) images of fibrous cap VSMCs in sham/*ApoE*^−/−^ and CKD/*ApoE*^−/−^ mice after giripladib treatment. Scale bars, 2 μm. (E) Representative images of α-SMA immunofluorescence (IF) staining in aortic root plaques of sham/*ApoE*^−/−^ and CKD/*ApoE*^−/−^ mice after giripladib treatment. Scale bar, 10 μm (hematoxylin and eosin [H&E]). (F) Quantification of the fold change of fibrous cap thickness and fibrous cap VSMCs (α-SMA^+^) contents in aortic root plaques of sham/*ApoE*^−/−^ and CKD/*ApoE*^−/−^ mice after giripladib treatment (*n* = 10). (G and H) Representative images and quantification of Oil Red O staining in arteries of sham/*ApoE*^−/−^ and CKD/*ApoE*^−/−^ mice after giripladib treatment (*n* = 10). (I to K) Representative H&E staining images, quantification of mean plaque fraction area and necrotic area percentage within plaques in sham/*ApoE*^−/−^ and CKD/*ApoE*^−/−^ mice after giripladib treatment (*n* = 10). Scale bar, 200 μm. Data represent means ± SD. **P < 0.01 and ***P < 0.001, ANOVA.

Given that cytosolic phospholipase A2 (cPLA2) is the major rate-limiting enzyme for releasing PUFA [21], we used a specific cPLA2 inhibitor, giripladib, to block PUFA accumulation [22]. Giripladib treatment ameliorated PUFA accumulation (Fig. S4A to C), reduced arterial MDA production, diminished 4-HNE expression, and mitigated mitochondrial-ferroptosis-associated morphological alterations in fibrous cap VSMCs within the atherosclerotic plaques of CKD mice (Fig. 3A to D). Furthermore, it significantly mitigated atherosclerotic plaque vulnerability (Fig. 3E to K and Fig. S4D and E). Consistent with these findings in vivo, pretreatment of hVSMCs with giripladib significantly attenuated CKD-serum-induced PUFA accumulation (Fig. S4F to H), inhibited the elevation of MDA, ROS, and lipid ROS levels, and restored the GSH/GSSG ratio in hVSMC (Fig. S4I to M). Overall, these data emphasize that PUFA accumulation promotes VSMC ferroptosis and CKD-associated atherosclerotic plaque vulnerability.

### *ECH1* deficiency contributes to PUFA accumulation and VSMC ferroptosis under CKD milieus

**Fig. 4.**
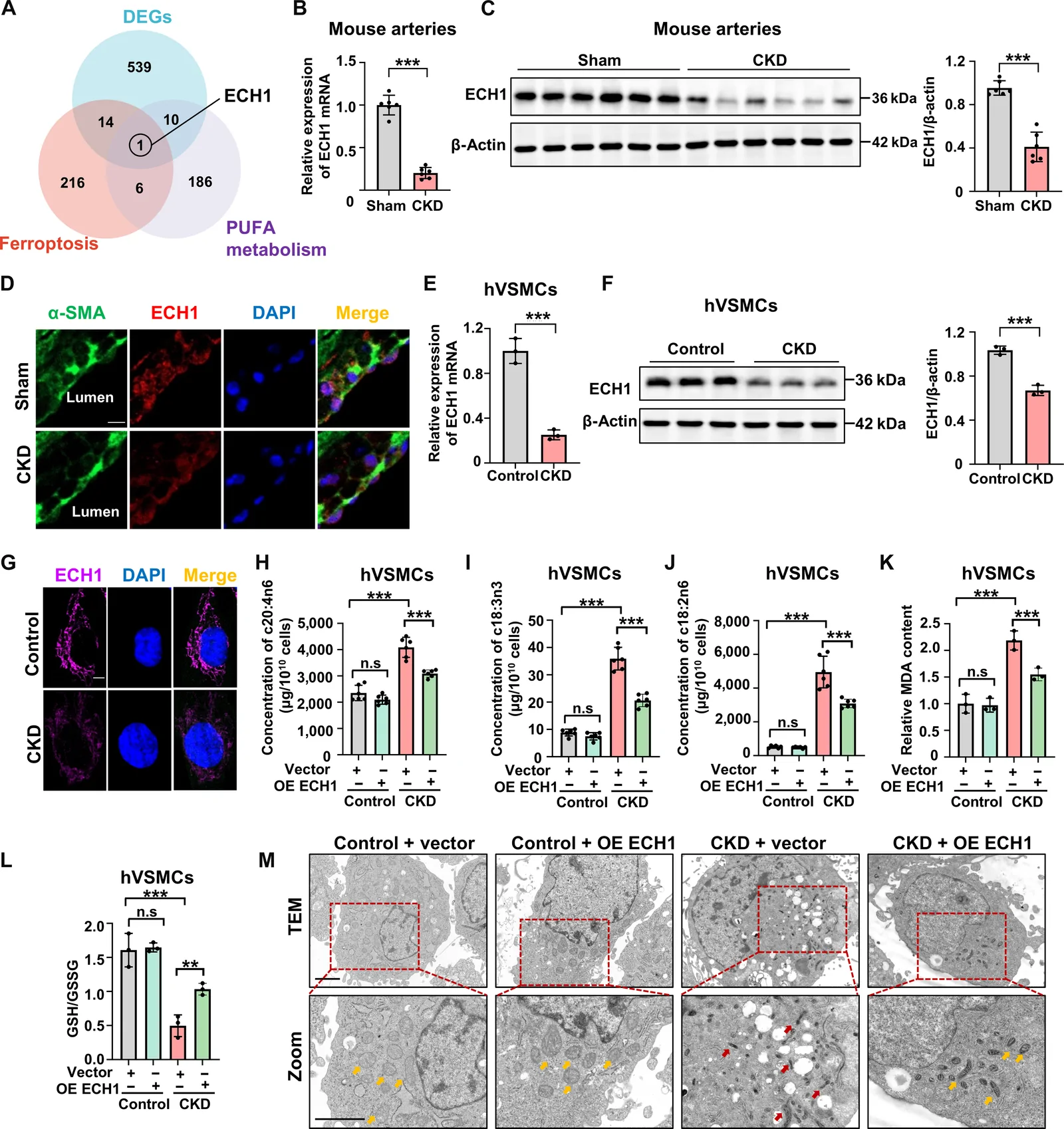
Enoyl-coenzyme A hydratase 1 (*ECH1*) deficiency is responsible for polyunsaturated fatty acid (PUFA) accumulation and vascular smooth muscle cell (VSMC) ferroptosis. (A) Venn diagram shows the intersection of differentially expressed genes (DEGs) from the GSE135626 microarray dataset, ferroptosis regulators, and PUFA metabolism-related genes. (B) Relative mRNA expression levels of *ECH1* in arteries of sham/*ApoE*^−/−^ and chronic kidney disease (CKD)/*ApoE*^−/−^ mice (*n* = 6). (C) Western blot (WB) analysis and quantification of ECH1 expression levels in arteries of sham/*ApoE*^−/−^ and CKD/*ApoE*^−/−^ mice (*n* = 6). (D) Representative images of *ECH1* and α-smooth muscle actin (α-SMA) staining in fibrous cap VSMCs of sham/*ApoE*^−/−^ and CKD/*ApoE*^−/−^ mice. Scale bar, 20 μm. (E) Relative mRNA expression levels of *ECH1* in human VSMCs (hVSMCs) after incubation with normal or CKD serum (*n* = 3). (F) WB analysis and quantification of *ECH1* expression levels in hVSMCs after incubation with normal or CKD serum (*n* = 3). (G) Representative images of *ECH1* staining in hVSMCs after incubation with normal or CKD serum. Scale bar, 5 μm. (H to J) Quantification of the level of arachidonic acid (AA), linoleic acid (LA), and linolenic acid (ALA) in hVSMCs incubated with normal or CKD serum after *ECH1* overexpression (OE) (*n* = 6). (K and L) Relative of malondialdehyde (MDA) content and the ratio of reduced glutathione (GSH)/oxidized glutathione (GSSG) in hVSMCs incubated with normal or CKD serum after *ECH1* overexpression (*n* = 5). (M) Representative transmission electron microscopy (TEM) images of VSMCs in hVSMCs incubated with normal or CKD serum after *ECH1* overexpression. Scale bars, 2 μm. Data represent means ± SD. **P < 0.01 and ***P < 0.001; Student’s *t* test was applied to (B-C and E-F); one-way analysis of variance (ANOVA) was applied to (H-L). n.s., not significant.

To investigate the underlying mechanisms of PUFA accumulation in VSMCs under CKD milieus, we integrated differentially expressed genes (DEGs) from previous microarray data (GSE135626), FerrDB databases [23], and genes implicated in PUFA metabolism by intersection analysis [24]. The results hinted that *ECH1* might be instrumental in PUFA accumulation and VSMC ferroptosis (Fig. 4A). Accordingly, the expression of *ECH1* was markedly decreased in arteries and fibrous cap VSMCs of CKD/*ApoE*^−/−^ mice and human vascular samples from patients with ESRD (Fig. 4B to D and Fig. S5A). In vitro, *ECH1* expression was also decreased in hVSMCs after CKD serum treatment (Fig. 4E to G). Overexpression of *ECH1* (Fig. S5B and C) in hVSMCs alleviated the accumulation of PUFAs and mitochondrial damage and suppressed the MDA, ROS, and lipid ROS levels, as well as the decreased ratio of GSH/GSSG, indicating a reduction in lipid peroxidation and ferroptosis (Fig. 4H to M and Fig. S5D to K).

*ECH1* primarily metabolizes endogenous PUFAs. Meanwhile, exogenous AA, a prototypical ω-6 PUFA, is oxidized by lipoxygenase to induce ferroptosis [25]. Thus, we further investigated whether exogenous AA could independently or synergistically promote ferroptosis in CKD-serum-incubated hVSMCs to simulate clinical scenarios of excessive PUFA exposure. We found that AA treatment exacerbated the accumulation of AA, MDA, ROS, and lipid ROS levels, as well as the decreased ratio of GSH/GSSG in CKD-serum-incubated VSMCs (Fig. S6A to J). However, AA treatment alone did not affect lipid peroxidation in VSMCs (Fig. S6A to J). Moreover, *ECH1* overexpression could reverse the exacerbating effect of AA on CKD-induced ferroptosis in VSMCs (Fig. S7A to E). Collectively, these results indicated that the down-regulation of *ECH1* in VSMCs induced by CKD milieus is a major factor in promoting PUFA accumulation, which subsequently triggers lipid peroxidation and ferroptosis.

### Human antigen R down-regulation induces *ECH1* deficiency in VSMCs under CKD milieus

**Fig. 5.**
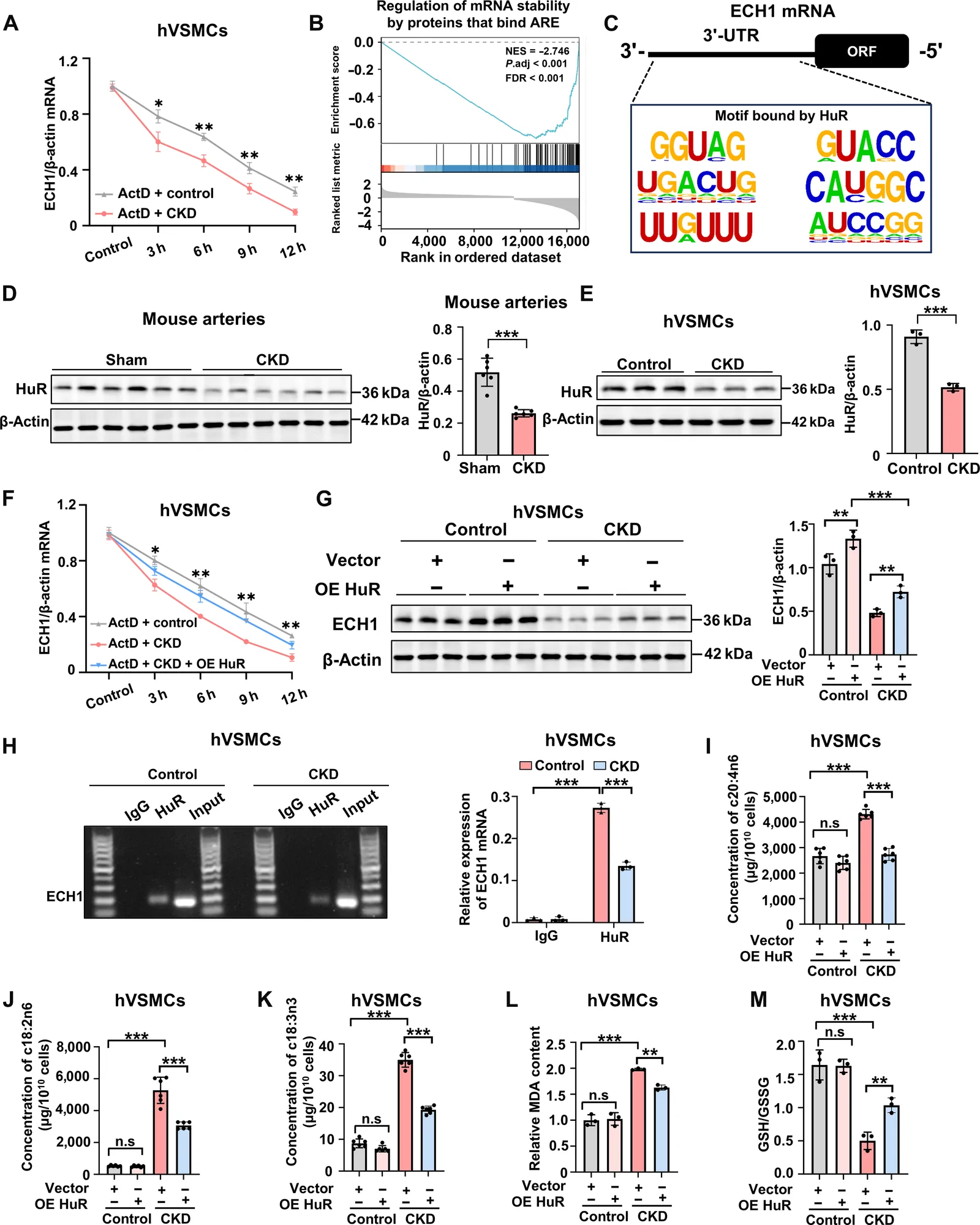
Human antigen R (HuR) down-regulation-induced enoyl-coenzyme A hydratase 1 (*ECH1*) deficiency in vascular smooth muscle cells (VSMCs) under chronic kidney disease (CKD) milieus. (A) Detection of mRNA stability of *ECH1* in human VSMCs (hVSMCs) after incubation with normal or CKD serum (*n* = 3). (B) Gene set enrichment analysis (GSEA) analysis of “Regulation of mRNA stability by proteins that bind ARE” gene set in aortic VSMCs of sham/*ApoE*^−/−^ and CKD/*ApoE*^−/−^ mice. (C) Predicted 6 motifs in the 3′-untranslated region (UTR) of *ECH1* mRNA bound by HuR. (D) Western blot (WB) analysis and RNA quantification of HuR expression levels in arteries of sham/*ApoE*^−/−^ and CKD/*ApoE*^−/−^ mice (*n* = 6). (E) WB analysis and quantification of HuR expression levels in hVSMCs after incubation with normal or CKD serum (*n* = 3). (F) Detection of mRNA stability of *ECH1* in hVSMCs normal or CKD serum after *ECH1* overexpression (*n* = 3). (G) WB analysis and quantification of ECH1 expression in hVSMCs after incubation with normal or CKD serum after HuR overexpression (*n* = 3). (H) RNA immunoprecipitation (RIP)-quantitative polymerase chain reaction (qPCR) of HuR and *ECH1* mRNA in hVSMCs after incubation with normal or CKD serum (*n* = 3). (I to K) Quantification of the level of arachidonic acid (AA), linoleic acid (LA), and linolenic acid (ALA) in hVSMCs incubated with normal or CKD serum after *HuR* overexpression (*n* = 6). (L and M) Relative of malondialdehyde (MDA) content and the ratio of reduced glutathione (GSH)/oxidized glutathione (GSSG) in hVSMCs incubated with normal or CKD serum after *HuR* overexpression (*n* = 3). *P < 0.05, **P < 0.01, and ***P < 0.001; Student’s *t* test (A), (D), and (E); ANOVA was applied to (F) to (M). ActD, actinomycin D; ORF, open reading frame.

To elucidate the underlying mechanism of *ECH1* deficiency, we detected the stability of *ECH1* mRNA in hVSMCs after CKD serum treatment and found a significant reduction (Fig. 5A). GSEA of previous microarray data revealed enrichment in the “Regulation of mRNA stability by proteins that bind ARE” pathway (Fig. 5B) [26]. Database predictions and previous cross-linking and immunoprecipitation sequencing data identified that human antigen R (HuR) is the only adenine–uracil-rich element (ARE)-binding protein predicted protein to interact with *ECH1* mRNA, and HuR binds to 6 motifs within the 3′-untranslated region (UTR) region of *ECH1* mRNA (Fig. 5C). Moreover, a positive correlation was observed between *ECH1* and *HuR* expression (http://utrdb.cloud.ba.infn.it/utrdb/). Notably, decreased HuR levels were observed in arteries of CKD/*ApoE*^−/−^ mice and human vascular samples from patients with ESRD (Fig. 5D and Fig. S8A) and CKD-serum-treated VSMCs in vitro (Fig. 5E). Subsequently, knocking down *HuR* using short interfering RNA in vitro (Fig. S8B and C) reduced the stability of *ECH1* mRNA, thereby decreasing the expression of *ECH1* (Fig. S8F and G). Overexpression of *HuR* (Fig. S6D and E) significantly mitigated CKD-induced *ECH1* mRNA instability and enhanced *ECH1* expression (Fig. 5F and G). Notably, RNA immunoprecipitation (RIP)-polymerase chain reaction (PCR) experiments confirmed a direct binding relationship between HuR and *ECH1* mRNA (Fig. 5H). Furthermore, overexpression of *HuR* mitigated CKD-induced accumulation of PUFAs, attenuated the elevation of MDA levels, and prevented the decline of GSH-to-GSSG ratio (Fig. 5I to M). Therefore, the above data suggest that HuR down-regulation serves as a critical mechanism underlying *ECH1* deficiency.

### VSMC-specific *ECH1* overexpression stabilizes atherosclerotic plaques by inhibiting ferroptosis in fibrous cap VSMCs

**Fig. 6.**
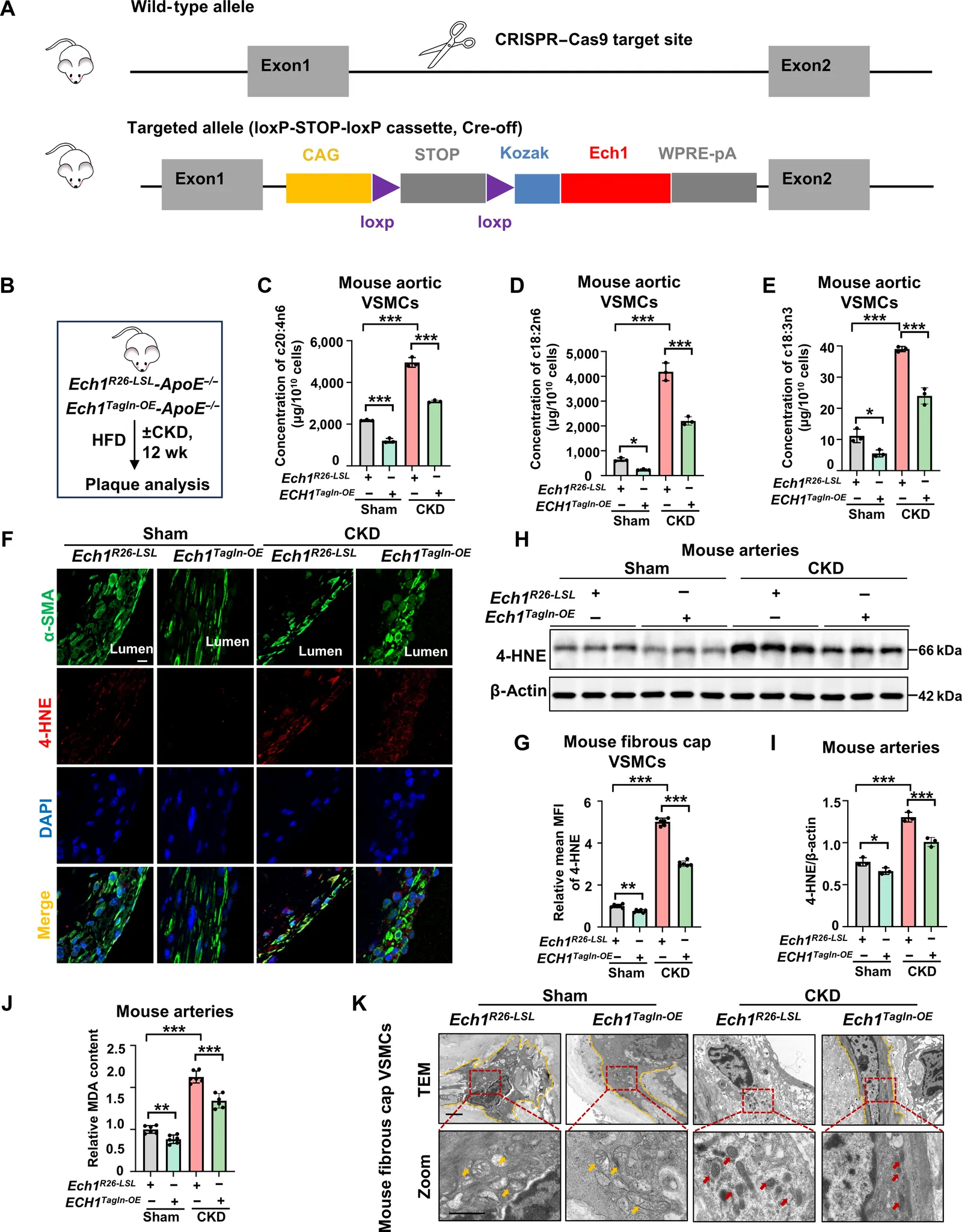
Vascular smooth muscle cell (VSMC)-specific overexpression of enoyl-coenzyme A hydratase 1 (*ECH1*) reduced ferroptosis of fibrous cap VSMCs. (A) Schematic illustration of the generation of *ECH1*^R26-LSL^ conditional knock-in mice via CRISPR–Cas9-mediated homology-directed repair. (B) The protocol of plaque analysis between *ECH1*^R26-LSL^-*ApoE*^−/−^ and *ECH1*^Tagln-OE^-*ApoE*^−*/*−^ mice with or without chronic kidney disease (CKD) operation. HFD, high-fat diet. (C to E) Quantification of the level of arachidonic acid (AA), linoleic acid (LA), and linolenic acid (ALA) in arteries of *ECH1*^R26-LSL^-*ApoE*^−/−^ and *ECH1*^Tagln-OE^-*ApoE*^−*/*−^ mice with or without CKD operation (*n* = 6). (F and G) Representative images and quantification of relative mean fluorescence intensity (MFI) of 4-hydroxynonenal (4-HNE) and α-smooth muscle actin (α-SMA) staining in fibrous cap VSMCs in arteries of *ECH1*^R26-LSL^-*ApoE*^−/−^ and *ECH1*^Tagln-OE^-*ApoE*^−/−^ mice with or without CKD operation (*n* = 6). Scale bar, 10 μm. (H and I) Western blot (WB) analysis and quantification of 4-HNE expression levels in arteries of *ECH1*^R26-LSL^-*ApoE*^−/−^ and *ECH1*^Tagln-OE^-*ApoE*^−/−^ mice with or without CKD operation (*n* = 3). (J) Relative of malondialdehyde (MDA) content in arteries of *ECH1*^R26-LSL^-*ApoE*^−/−^ and *ECH1*^Tagln-OE^-*ApoE*^−/−^ mice with or without CKD operation (*n* = 6). (K) Representative transmission electron microscopy (TEM) images of fibrous cap VSMCs in *ECH1*^R26-LSL^-*ApoE*^−/−^ and *ECH1*^Tagln-OE^-*ApoE*^−/−^ with or without CKD operation. Scale bar, 2 μm. Data represent means ± SD. **P < 0.05, **P < 0.01, and ***P < 0.001, ANOVA.

**Fig. 7.**
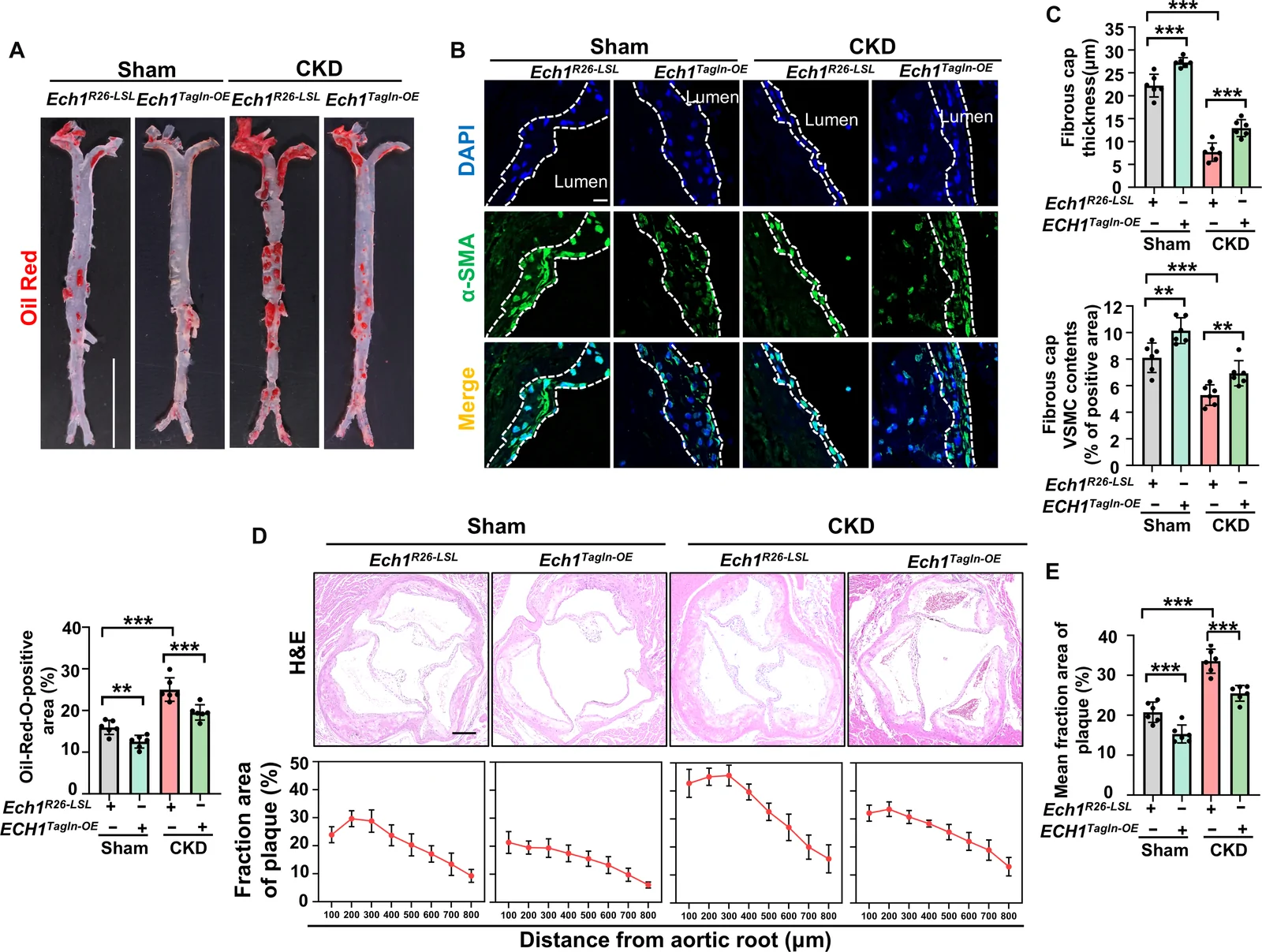
Vascular smooth muscle cell (VSMC)-specific overexpression of enoyl-coenzyme A hydratase 1 (*ECH1*) mitigates atherosclerotic plaque vulnerability. (A) Representative images and quantification of Oil Red O staining in arteries of sham/*ApoE*^−/−^ and chronic kidney disease (CKD)/*ApoE*^−/−^ mice after *ECH1* overexpression (*n* = 10). Scale bar, 2 μm. (B) Representative images of α-smooth muscle actin (α-SMA) immunofluorescence (IF) staining in aortic root plaques of sham/*ApoE*^−/−^ and CKD/*ApoE*^−/−^ mice after *ECH1* overexpression. Scale bar, 10 μm (hematoxylin and eosin [H&E]). (C) Quantification of the fold change of fibrous cap thickness and fibrous cap VSMCs (α-SMA^+^) contents in aortic root plaques of sham/*ApoE*^−/−^ and CKD/*ApoE*^−/−^ mice after *ECH1* overexpression (*n* = 10). (D and E) Representative H&E staining and quantification of the fraction area of multiple sections of aortic root plaques in sham/*ApoE*^−/−^ and CKD/*ApoE*^−/−^ mice after *ECH1* overexpression (*n* = 10). Scale bar, 200 μm. **P < 0.01 and ***P < 0.001, ANOVA.

To further elucidate the role of *ECH1* in vulnerable atherosclerotic plaque, we established a mouse model featuring a conditional site-specific knock-in of *ECH1* at the Rosa26 locus [C57BL/6J-Gt(ROSA)26Sor^em1(CAG-LSL-Kozak-*ECH1*-WPRE-pA)1Smoc^] (Fig. 6A and Table S1). Subsequently, this mouse strain was crossed with Tagln-Cre and *ApoE*^−/−^ mouse to generate *ApoE*^−/−^ mouse exhibiting VSMC-specific overexpression of *ECH1* (*ECH1*^Tagln-OE^-*ApoE*^−/−^) (Fig. 6B and Fig. S9A to C). *ECH1*^R26-LSL^-*ApoE*^−/−^ mice were applied as the control group. *ECH1*^Tagln-OE^-*ApoE*^−/−^ and *ECH1*^R26-LSL^-*ApoE*^−/−^ mice underwent either a sham operation or CKD surgery and were subsequently maintained on a high-fat diet for 12 weeks (Fig. 6B). Notably, VSMC-specific overexpression of *ECH1* ameliorated PUFA accumulation, decreased the expression of 4-HNE and MDA, and suppressed mitochondrial damage in plaque fibrous cap VSMCs (Fig. 6C to K). Meanwhile, VSMC-specific overexpression of *ECH1* markedly attenuated atherosclerosis progression and enhanced atherosclerotic plaque stability (Fig. 7A to E and Fig. S10A to D). In conclusion, these findings suggested that *ECH1*-deficiency-induced VSMC ferroptosis promotes plaque vulnerability and atherosclerosis progression in mice, especially in CKD/*ApoE*^−/−^ mice.

### Inhibition of ACSL4 alleviates VSMC ferroptosis and atherosclerotic plaque vulnerability of CKD/*ApoE*^−/−^ mice

**Fig. 8.**
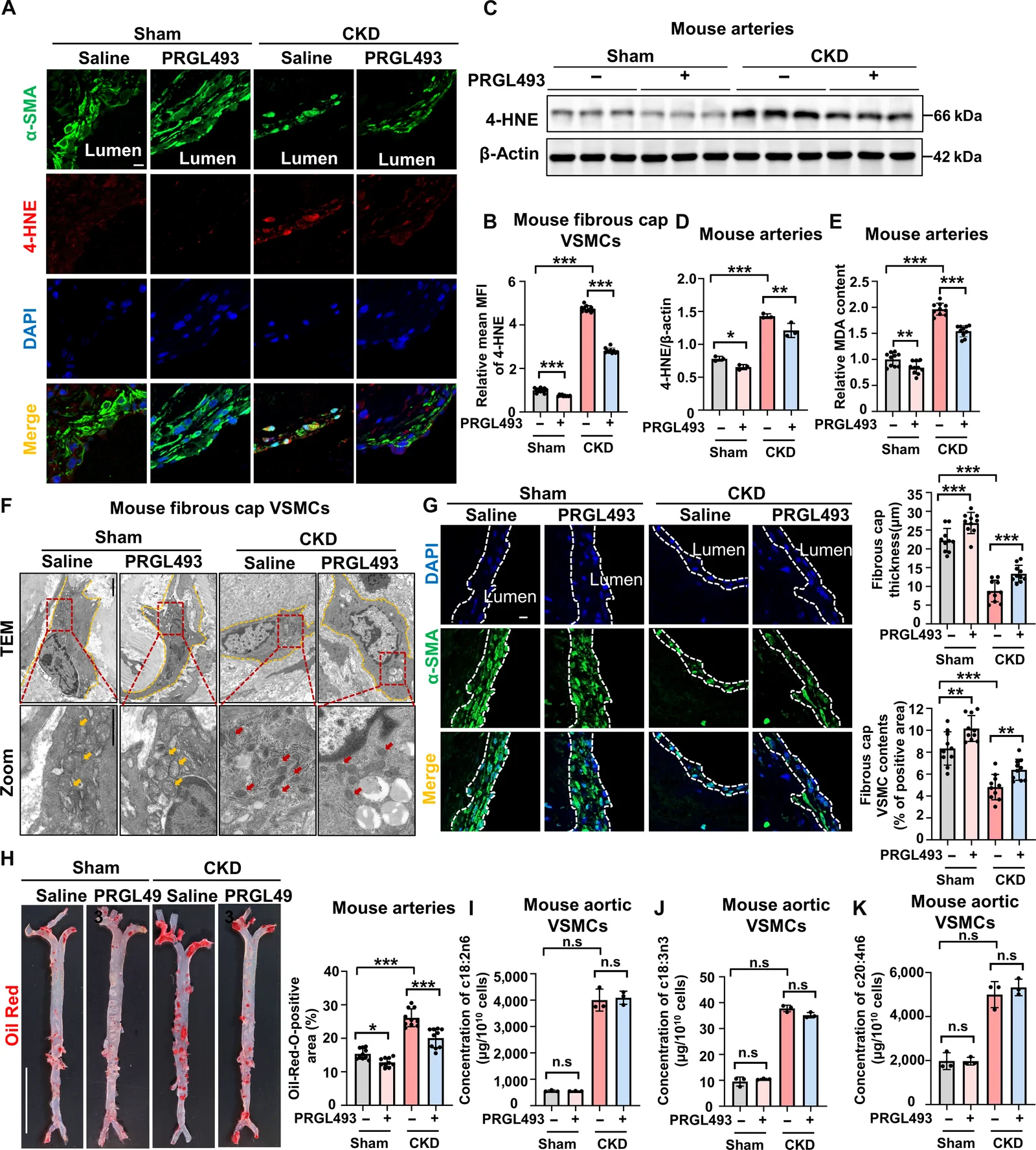
Inhibition of acyl-coenzyme A synthetase long-chain family member 4 (ACSL4) alleviates vascular smooth muscle cells (VSMC) ferroptosis and atherosclerotic plaque vulnerability of chronic kidney disease (CKD)/*ApoE*^−/−^ mice. (A and B) Representative images and quantification of relative mean fluorescence intensity (MFI) of α-smooth muscle actin (α-SMA) and 4-hydroxynonenal (4-HNE) staining in fibrous cap VSMCs of sham/*ApoE*^−/−^ and CKD/*ApoE*^−/−^ mice after PRGL493 treatment (*n* = 10). Scale bar, 10 μm. (C and D) Western blot (WB) analysis and quantification of 4-HNE expression levels in arteries of sham/*ApoE*^−/−^ and CKD/*ApoE*^−/−^ mice after PRGL493 treatment. (E) Relative of malondialdehyde (MDA) content in arteries of sham/*ApoE*^−/−^ and CKD/*ApoE*^-/−^ mice after PRGL493 treatment (*n* = 10). (F) Representative transmission electron microscopy (TEM) images of fibrous cap VSMCs in sham/*ApoE*^−/−^ and CKD/*ApoE*^−/−^ mice after PRGL493 treatment. Scale bar, 2 μm. (G) Representative images of α-SMA immunofluorescence (IF) staining in aortic root plaques of sham/*ApoE*^−/−^ and CKD/*ApoE*^−/−^ mice after PRGL493 treatment. Scale bar, 10 μm. Quantification of the fold change of fibrous cap thickness and fibrous cap VSMCs (α-SMA^+^) contents in aortic root plaques of sham/*ApoE*^−/−^ and CKD/*ApoE*^−/−^ mice after PRGL493 treatment (*n* = 10). (H) Representative images and quantification of Oil Red O staining in arteries of sham/*ApoE*^−/−^ and CKD/*ApoE*^−/−^ mice after PRGL493 treatment. Scale bar, 2 μm. (*n* = 10). (I to K) Quantification of the level of arachidonic acid (AA), linoleic acid (LA), and linolenic acid (ALA) in arteries of sham/*ApoE*^−/−^ and CKD/*ApoE*^−/−^ mice after PRGL493 treatment (*n* = 6).

Previous studies have demonstrated that acyl-coenzyme A synthetase long-chain family member 4 (ACSL4) is a key enzyme in PUFA lipid peroxidation [27]. Thus, the pharmacological ACSL4 inhibitor PRGL493 was used to explore the role of PUFA metabolism in VSMC ferroptosis within vulnerable atherosclerotic plaques. PRGL493 treatment decreased the expression of 4-HNE in fibrous cap VSMCs and arteries (Fig. 8A to D), reduced arterial MDA production, and attenuated the characteristic features of ferroptosis in fibrous cap VSMCs in CKD/*ApoE*^−/−^ mice (Fig. 8E and F). Consistent with these findings in vivo, PRGL493 pretreatment significantly mitigated mitochondrial damage, reduced MDA, ROS, and lipid ROS levels, and restored the GSH/GSSG ratio in CKD-serum-incubated hVSMCs (Fig. S11D to K). Nonetheless, there was no significant impact of PRGL493 on PUFA levels (Fig. 8I to K and Fig. S11A to C). Furthermore, PRGL493 treatment markedly mitigated atherosclerosis progression and plaque vulnerability (Fig. 8G and H and Fig. S12A to D).

At last, as PRGL493 and *ECH1* overexpression mitigated CKD-induced ferroptosis via distinct mechanisms, we further explored the potential synergistic inhibitory effects of combining PRGL493 with *ECH1* overexpression. Combined treatment exhibited a significantly enhanced synergistic inhibitory effect on CKD-induced hVSMC ferroptosis and resulted in a marked reduction in ferroptosis-associated markers (including MDA, ROS, and lipid ROS levels in hVSMCs) (Fig. S13A to D), accompanied by a significant increase in GSH/GSSG ratio (Fig. S13E).

## Discussion

Recent studies have demonstrated that stable and vulnerable plaques exhibit distinct spatial distribution characteristics of lipids across different plaque regions, suggesting that investigating local lipid profile could provide a novel perspective for exploring molecular mechanisms of atherosclerosis progression [28]. However, research in this field currently relies predominantly on untargeted spatial metabolomics techniques [29,30], which have inherent limitations. Certain lipid molecules (such as PUFAs and MUFAs) are prone to degradation at plaque sites during detection [31], resulting in loss of critical lipid information. Notably, targeted spatial metabolomics studies specifically focusing on the fibrous cap region of atherosclerotic plaques remain unexplored. This study bridges this gap through integrated multidimensional approaches. First, single-cell analysis of vascular samples from patients with CKD revealed distinct lipid metabolic dysregulation signatures in VSMCs under CKD milieus. Subsequent in vitro targeted metabolomics validation confirmed that CKD induces significant accumulation of PUFAs in VSMCs. Finally, targeted spatial metabolomics techniques clarified that PUFAs specifically accumulate in the fibrous cap region of CKD-associated plaques. These findings provide pivotal molecular evidence for understanding the lipid metabolic mechanisms driving CKD-related plaque vulnerability.

The stability of atherosclerotic plaques is a critical factor influencing the prognosis in patients with cardiocerebrovascular diseases. VSMCs, as the principal structural component of the fibrous cap in atherosclerotic plaques, play an essential role in regulating atherosclerotic plaque stability [7]. Growing evidence indicates that VSMC ferroptosis is closely associated with multiple cardiovascular diseases [32], including vascular calcification [33], aortic dissection [34], abdominal aortic aneurysm [35], and atherosclerosis [36]. Notably, 2 studies have directly examined the impact of VSMC ferroptosis in plaque stability. One study finds that administration of ferroptosis inhibitor ferrostatin-1 (1.0 mg/kg) stabilizes atherosclerotic plaques in *ApoE*^−/−^ mice [37]. Nonetheless, this study uses a conventional model of atherosclerosis in *ApoE*^−/−^ mice. Another study established an atherosclerosis model in *ApoE*^−/−^ mice by subjecting them to partial ligation of the left common carotid artery, followed by 4 weeks of high-fat diet feeding [38]. This model is characterized by rapid progression and localized lesion formation [39]. In contrast to the model utilized in our study, it is noteworthy that CKD/*ApoE*^−/−^ mouse model approximates more closely the complex pathological context observed in humans, while the former model lacks the systemic metabolic disease background. The establishment and merits of CKD/*ApoE*^−/−^ mouse model have been comprehensively examined in our previously published work [11]. Thus, above evidence suggests that inhibiting VSMC ferroptosis may be a potential therapeutic approach for the treatment of atherosclerosis progression and vulnerable plaques.

Accumulating evidence indicates that ferroptosis contributes to the pathogenesis of multiple diseases [40,41]. Previous studies have explored the molecular mechanisms of VSMC ferroptosis in cardiovascular disease, focusing on factors such as GSH/GPX4 inhibition [42], iron metabolic dysfunction [34], oxidative stress [43], mitochondrial dysfunction [44], and epigenetic regulation [45]. While PUFA metabolism is recognized as a crucial mechanism in ferroptosis [46], its specific role and regulatory mechanism in VSMCs within atherosclerotic vulnerable plaque remain inadequately understood. Through spatial and targeted metabolomics and gas chromatography-mass spectrometry (GC-MS) detection, we examine long-chain fatty acid levels in VSMCs and discover that PUFA accumulation induces VSMC ferroptosis. Our findings underscore the pivotal role of PUFA metabolism. Previously, impaired catabolism and accumulation of PUFA have been identified as critical mediators of ferroptosis in prostate cancer cells [47]. Studies [48,49] have demonstrated that silencing 2,4-dienoyl-CoA reductase 1 (DECR1), a key gene involved in PUFA metabolism, disrupts normal PUFA metabolic processes. This disruption leads to the intracellular accumulation of PUFA, thereby triggering lipid peroxidation and ultimately promoting the onset of ferroptosis. Similarly, our findings reveal that PUFA metabolic dysfunction and accumulation constitute central mechanisms driving VSMC ferroptosis in vulnerable atherosclerotic plaque.

ECH1 is an enzyme predominantly localized within the mitochondria and facilitates the catabolism of PUFA as energy-producing fatty acid substrates [50]. Consequently, the expression and activity of ECH1 are critical for the rate and efficiency of PUFA β-oxidation [51]. Notably, endogenous *ECH1* has been reported to suppress ferroptosis in liver cells [52]. However, the precise mechanism by which *ECH1* inhibits ferroptosis remains inadequately explored. In this study, we discover the mechanism through which *ECH1* suppresses ferroptosis induced by PUFA accumulation. Previous research has examined the effects of exogenous *ECH1* on atherosclerotic plaques, revealing that supplementation with ECH1 recombinant protein (0.5 μg/ml) inhibited plaque formation in *ApoE*^−/−^ mice [53]. Nonetheless, this study did not investigate the impact of endogenous *ECH1* in VSMCs on plaque stability. In our study, we further elucidate the role of endogenous *ECH1*. By developing a VSMC-specific *ECH1* overexpression mouse model and overexpressing *ECH1* in vitro, we demonstrate that overexpression of endogenous *ECH1* inhibits VSMC ferroptosis and improves plaque stability.

HuR regulates posttranscriptional processes by binding to specific mRNA sequences, exerting central regulatory roles in tumorigenesis [54], inflammation, and vascular diseases [55]. Recent studies have revealed that HuR exhibits a dual functional mode in atherosclerosis progression [56]. It has been reported that HuR enhances C-FOS protein levels by stabilizing C-FOS mRNA, directly driving VSMC proliferation and migration, and promoting vascular remodeling and neointima hyperplasia, which is a key driving step in early atherosclerosis pathogenesis [57]. Liu et al. [58] confirmed that VSMC-specific knockout of *HuR* accelerates AMP-activated protein kinase catalytic subunit α1/α2 (AMPKα1/α2) mRNA degradation, induces autophagy deficiency, and exacerbates atherosclerotic instability. This aligns with our finding that HuR plays a protective role in maintaining plaque stability under CKD milieus. Vascular remodeling and neointima hyperplasia are early driving events in atherosclerosis [59], while in late-stage plaques, the main pathological characteristics shift to cell death, aging, and plaque vulnerability [9]. Thus, biological effects of HuR are determined by key mRNA targets and specific pathological microenvironments. Notably, we discover a novel interaction that HuR regulates *ECH1* mRNA stability in VSMCs and emphasize that targeting HuR for disease intervention requires precise analysis of cell type specificity and pathological-environment-dependent functional networks.

Given the critical involvement of ferroptosis in VSMCs within atherosclerotic vulnerable plaque, we evaluate the translational potential of our study in treating vulnerable plaque using newly identified cPLA2 and ACSL4 inhibitors. Pharmacological inhibition of cPLA2 and ACSL4 inhibitors can mitigate the release of PUFA and correspondingly relieve PUFA lipid peroxidation. Previous studies have shown that traditional Chinese medicinal compounds with cPLA2 inhibitory activity, such as tanshinone I [60] and salidroside [61], can significantly decrease the risk of cardiovascular disease. Our findings indicate that giripladib, a specific cPLA2 inhibitor, [62] exerts a protective role on vulnerable atherosclerotic plaque. However, clinical trials of giripladib are terminated at phase II (clinicaltrials.gov) due to pronounced gastrointestinal side effects. Therefore, future research on cPLA2 inhibition for treating vulnerable plaques could focus on the active ingredients in traditional Chinese medicine and the structural modifications of giripladib. ACSL4 serves as a crucial enzyme in the process of PUFA lipid peroxidation [63]; research has shown that its activity can be inhibited through various pathways. Inhibition of ACSL4 with atorvastatin [64], berberine [65], or magnesium ions [66] could significantly retard the atherosclerotic progression. In addition, recent studies have identified PRGL493 as a highly efficient and selective inhibitor of ACSL4 [67]. However, the application of PRGL493 in atherosclerosis-related research has not yet been explored, we demonstrate that PRGL493 plays a protective role in vulnerable atherosclerotic plaque.

The limitation of this study is that we only characterized the accumulation of AA, LA, and ALA, while the profiles of ω-3 PUFAs (e.g., eicosapentaenoic acid, docosahexaenoic acid, and docosapentaenoic acid) were not systematically examined. The potential regulatory effects of ω-3 PUFAs on VSMC ferroptosis and atherosclerotic lesion progression deserve independent and thorough investigation in future work. Moreover, while we delineate the downstream ECH1/HuR regulatory axis underlying PUFA accumulation, the specific upstream pathogenic mediators in CKD serum that initiate this metabolic reprogramming remain to be definitively isolated and functionally validated.

In conclusion, our study demonstrates that *ECH1* deficiency in fibrous cap VSMCs induced PUFA accumulation and enhanced lipid peroxidation, which promote VSMC ferroptosis and contribute to the vulnerability of atherosclerotic plaques. Mechanically, the decreased expression of *ECH1* is mediated by the reduction of HuR. The application of giripladib, PRGL493, and overexpressing *ECH1* may provide promising therapeutic strategies by targeting PUFA metabolism in VSMCs for treating atherosclerotic plaque vulnerability. Overall, our research elucidates novel mechanisms underlying atherosclerotic plaque vulnerability and suggests promising strategies for its prevention and treatment.

## Materials and Methods

### Animals

*ApoE*^−/−^ mice (C57BL/6J background, male, 8 weeks old, weighing 20 to 25 g) were obtained from SJA Laboratory Animal Co. Ltd. C57BL/6J-Gt(ROSA)26Sor^em1(CAG-LSL-Kozak-*ECH1*-WPRE-pA)1Smoc^ (*ECH1*^R26-LSL^) mice were generated using the CRISPR–Cas9 technique by the Shanghai Model Organisms Center. Specifically, the CRISPR–Cas9 technology was used to introduce a knock-in at the Rosa26 locus. This technology involves designing guide RNAs specific to the gene of interest, which direct the Cas9 nuclease to modify the genome at the insertion site. This process enhances the efficiency of homologous recombination within the targeted genomic region, facilitating the homologous recombination of the target fragment into the desired locus. The inserted coding DNA sequence has a full length of 984 bp, with the specific sequence detailed in Table S1. To generate *ECH1*^Tagln-OE^-*ApoE*^−/−^ mice, *ECH1*^R26-LSL^ mice were crossed onto *ApoE*^−/−^ and Tagln-Cre mice. To compare plaque morphology, *ECH1*^R26-LSL^-*ApoE*^−/−^ mice were utilized as control. A classic 2-step 5/6 nephrectomy was performed to establish the CKD mouse model, with the detailed and standardized surgical procedure fully validated and described in our previous study [11]. After the second surgery, high-fat diet (H10141, HFK Biologic Technology) was administered to the mice. For giripladib (MedChemExpress) or PRGL493 (MedChemExpress) treatment, mice were treated utilizing a dose of 7.5 or 0.25 mg/kg intraperitoneally every day. After 12 weeks on the high-fat diet, the mice were anesthetized and euthanized; blood, hearts, and arteries were collected for further analyses. The metabolic parameters of mice are listed in Tables S2 to S5. All mice were pathogen-free and maintained under specific-pathogen-free conditions. Experimental units were individual mice. All the experimental procedures were approved by the Animal Care Committee of the Army Medical University (AMUWEC20222973).

### Single-cell RNA sequencing on 10x Genomics Chromium platform

Vascular tissues trimmed during surgery from 4 patients with ESRD scheduled for renal transplantation were collected as the disease group, while vascular tissues trimmed from 3 healthy donor kidneys were selected as the control group. Detailed baseline clinical characteristics of all enrolled subjects are summarized in Table S10. Subsequently, single-cell sample preparation of the vascular tissues was carried out, followed by cell counting and quality control to ensure that the samples contained an optimal concentration of viable cells. Based on the predetermined number of cells to be acquired, Gel Bead-in-Emulsions for single-cell separation were constructed. After the Gel Bead-in-Emulsions were normally formed, they were collected and placed in a PCR instrument for reverse transcription to achieve labeling. Then, cDNA amplification was performed, followed by the construction of a gene expression library. Ultimately, the library mixture was sequenced on an Illumina 10x Genomics Chromium sequencer using 150-bp paired-end reads. After quality control of the sequencing data, the remaining cells were normalized, and variable genes were identified. The Uniform Manifold Approximation and Projection (UMAP) method was used to visualize data in a 2-dimensional space. For cell type classification, the FindClusters function in Seurat was used for unsupervised clustering, which adopts a graph-based clustering approach. Based on the expression of marker genes, the resolved cell clusters were annotated as specific cell types. In addition, GO functional enrichment analysis and ferroptosis-associated gene set variation analysis were performed on the differential genes of VSMCs between control and CKD group. The study protocol was approved by the Ethics Committee of Xinqiao Hospital (2024-Research063-02).

### Cell culture

hVSMCs were acquired from the American Type Culture Collection. The culture condition is the Dulbecco’s modified Eagle’s medium (DMEM; Gibco) supplemented with 10% fetal bovine serum (Gibco) at 37 °C with 5% CO_2_. The modeling method for simulating the CKD internal environment in vitro using hVSMCs is the same as depicted above. We utilized hVSMCs from passages 2 to 5 for tests. Cells were cultured in DMEM containing 10% uremic serum or human normal serum. For drug administration, the following reagents were used before treating with human normal or uremic serum: 10 μM giripladib (MedChemExpress), 50 μM PRGL493 (MedChemExpress), and 2.5 μM AA (MedChemExpress).

### Transmission electron microscopy

Mouse aortas and hVSMCs were collected and fixed in 2.5% glutaraldehyde. The specimens were sent to the Biomedical Analysis Center (Army Medical University) for transmission electron microscopy (TEM) ultrastructural analysis. Using a JEM-1400 transmission electron microscope (JEOL, Tokyo, Japan) to examine and photograph the sections.

### GSH/GSSG assay

Based on the manufacturer’s procedures for the GSH/GSSG assay kit (Beyotime), intracellular GSH and GSSG levels were determined as follows. After treatment, hVSMCs were gathered, and the supernatant was removed. A protein removal reagent solution M was added, and the mixture was centrifuged at 10,000*g* for 10 min at 4 °C. Then, the supernatant was collected for analysis. The total glutathione content in each sample was determined by preparing a standard curve and measuring its absorbance at 412 nm. Subsequently, GSH in the samples was eliminated using a GSH-removing auxiliary solution, and absorbance at 412 nm was determined again to quantify GSSG levels. The GSH concentration was determined by subtracting the measured GSSG level from the total glutathione pool.

### MDA assay

The relative MDA concentrations in arterial tissues and hVSMCs were assessed using an MDA assay kit (Beyotime) based on the protocols. Arterial tissues or cell samples were homogenized and lysed using an immunoprecipitation lysis buffer. The lysates were then centrifuged at 10,000*g* for 10 min, and supernatants were collected for subsequent assays. After sample preparation, protein concentrations were quantified. Standard solutions, test samples, and detection working solution were added separately into a 96-well plate. Measuring the absorbance of each sample was at 532 nm with a microplate reader.

### Plaque and intracellular ROS analysis

To detect ROS production in plaques, fresh-frozen sections of the aortic root were incubated with 10 μM dihydroethidium (Thermo Fisher Scientific) dissolved in phosphate-buffered saline (PBS) at 37 °C for 0.5 h in the dark. The sections were immediately photographed utilizing a Zeiss LSM800 NLO confocal microscope (Carl Zeiss), and fluorescence intensity was quantified via ZEN software (Carl Zeiss).

### Western blot analyses

Protein specimens isolated from hVSMCs or endothelium-removed aortas were used for Western blotting analysis, as previously described. Briefly, protein specimens were separated by SDS–polyacrylamide gel electrophoresis and transferred onto polyvinylidene difluoride membranes (Millipore). The membranes were blocked with 5% skimmed milk powder for 2 h at 37 °C and subsequently incubated overnight at 4 °C utilizing primary antibodies. Primary antibody against 4-HNE (ab48506; 1:1,000) was bought from Abcam (Cambridge, MA, UK). The primary antibodies against ECH1 (A12944; 1:1,000), HuR (A19622; 1:1,000), GPX4 (A27995; 1:1,000), SLC7A11(A25302; 1:1,000), and β-actin (AC026; 1:1,000) were obtained from Abclonal (Wuhan, China). β-Actin was used as loading control for normalization. Then, the membranes were incubated with secondary antibodies for 1 h, and signals were detected using a Bio-Rad ChemiDoc MP imager (Bio-Rad Laboratories, Hercules, CA, USA).

### Quantitative PCR

RNA was extracted from endothelium-removed aortas and hVSMCs using TRIzol Plus reagent (TaKaRa) and reverse-transcribed into cDNA using PrimeScript RT Reagent Kit (TaKaRa). Quantitative PCR (qPCR) was performed on the CFX96 Real-Time System (Bio-Rad) using the GoTaq qPCR Master Mix (Promega) to determine the mRNA expression levels of the indicated genes. Data were normalized relative to β-actin. The primer sequences used are listed in Table S7.

### Oil Red O staining

The intact aorta was meticulously dissected from the aortic root to the iliac bifurcation, with all perivascular adipose and connective tissues carefully removed. The vessel was then longitudinally incised along the lesser curvature of the aortic arch to expose the lumen, extending proximally to the common carotid and subclavian arteries and distally to the iliac arteries. The dissected aorta was fixed in 4% paraformaldehyde for 24 h and subsequently stained with a freshly prepared Oil Red O working solution (Solarbio) at 37 °C for 60 min. Nonspecific background staining was differentiated by briefly rinsing the samples with 60% isopropanol. Quantitative analysis of the atherosclerotic lesion areas was conducted using ImageJ software.

### Histology analysis of atherosclerotic plaque

We analyzed atherosclerotic plaque morphology and composition as depicted above. Briefly, mouse hearts were perfused and fixed in 4% paraformaldehyde for 24 h before sectioning. The aortic roots were embedded in paraffin and serially sectioned at a thickness of 4 μm through the region of the aortic valves. Aortic root paraffin sections were stained with hematoxylin and eosin (H&E) (for plaque and necrotic core area analysis) and Masson’s trichrome (for collagen content quantification) following standard procedures and subjected to immunohistological staining using an anti-α-smooth muscle actin (α-SMA; Abcam). The stained tissues and sections were imaged utilizing a microscope (Olympus Optical), and quantitative analyses were performed using ImagePro software.

### FeCl_3_-induced carotid artery thrombosis model

Carotid arterial thrombosis was induced using the FeCl_3_ injury approach with minor modifications. Following adequate anesthesia, a midline cervical incision was made to carefully blunt-dissect and expose the common carotid artery. A perivascular miniature Doppler flow probe (Transonic Systems Inc.) was secured around the isolated vessel to continuously record real-time blood flow dynamics. To induce standardized endothelial injury, a 2 mm × 2 mm piece of Whatman filter paper presoaked with 10% FeCl_3_ solution was topically applied to the adventitial surface of the carotid artery for 60 s. After removal of the filter paper, the injured segment was thoroughly rinsed with prewarmed (37 °C) sterile saline, and blood flow was monitored continuously for a total of 30 min. Time to complete vessel occlusion was defined as the interval from FeCl_3_ application to persistent blood flow arrest lasting more than 30 s; specimens that did not develop full occlusion within the observation window were assigned a cutoff value of 30 min.

### Immunofluorescence

For immunofluorescence (IF) staining, paraffin sections were prepared as described above. Antigen retrieval was performed by boiling the sections in citrate buffer (Santa Cruz Biotechnology) at over 95 °C for 0.5 h for sections. Subsequently, the sections were blocked utilizing goat serum (Beyotime) for 30 min at room temperature. The sections were then incubated overnight at 4°C with primary antibodies against 4-HNE (Abcam), ECH1 (Abclonal), HuR (Abclonal), or α-SMA (Abcam). After incubation, appropriate fluorescent-dye-conjugated secondary antibodies were used and using 4′,6-diamidino-2-phenylindole (DAPI; Beyotime) to counterstain. Finally, sections were photographed using a Zeiss LSM800 NLO confocal microscope (Carl Zeiss).

### Ferroptosis assay

For the assessment of ferroptosis, fluorescent probes including 2′,7′-dichlorodihydrofluorescein diacetate (H_2_DCFDA) and Liperfluo were used to measure ROS and lipid peroxide levels in hVSMCs. Probe loading was performed either in situ or after cell collection, followed by confocal fluorescence microscopy imaging and flow cytometric analysis of the aforementioned indicators. To visualize the staining of the fluorescent probes, hVSMCs were incubated with 1 μM 2′,7′-dichlorodihydrofluorescein diacetate (MedChemExpress), 5 μM BODIPY 581/591 C11 (Beyotime), or 5 μM Liperfluo (Dojindo Molecular Technologies) at 37 °C for 30 min in the dark after treatment. Subsequently, hVSMCs were rinsed with PBS to remove extra probes and photographed using a Zeiss LSM800 NLO confocal microscope (Carl Zeiss). For flow cytometry analysis, cells were first digested with trypsin and then incubated with the fluorescent probes and under the same incubation conditions mentioned above. Following washing with PBS to remove unbound probes, the hVSMCs were analyzed by flow cytometry.

### Spatial targeted lipidomics

Spatial lipidomics enables the mapping of lipidomics distributions within aortic root plaque sections. Here, we present a targeted spatial metabolomics workflow that integrates on-tissue MS/MS fragmentation with authentic standard comparison for confident metabolite annotation. After pretreating the samples, which were stored at −80 °C, at 20 °C for 1 h, 10-μm sections were cut using a Leica CM1950 cryostat and immediately mounted onto indium-tin-oxide-coated slides (Bruker) for matrix-assisted laser desorption/ionization MS imaging; adjacent sections were stained with H&E. The slides were then vacuum-dried for 15 min, sealed, and stored at −80°C. A 1.5-mM 1,5-diaminonaphthalene solution (90% methanol/0.3% trifluoroacetic acid) was sprayed in 10 cycles using a TM-Sprayer (HTX Technologies) at the following parameters: speed, 1,200 mm/min; spacing, 3 mm; flow rate, 0.08 ml/min; temperature, 60 °C; nitrogen pressure, 10 psi. Images were acquired in negative ion mode using a Bruker thermal ionization mass spectrometry time-of-flight flex matrix-assisted laser desorption/ionization 2 with a 10-kHz SmartBeam 3-dimensional laser (range, 50 to 1,300 mass/charge ratio; pixel size, 10 μm × 10 μm; laser frequency, 10 kHz; energy, 70%; 200 repetitions per pixel). The data and ion imaging were processed using SCiLS Lab 2024b Pro. For targeted analysis, we prepared 3 standard samples in this experiment; details refer to the Table S8. Precursor ions of interest were selected on the basis of accurate mass (<3 parts per million), and on-tissue MS/MS spectra were acquired using collision energy ramping from 10 to 40eV to generate rich fragmentation patterns. Authentic standards were spotted onto metal target plate or the same tissue section at defined coordinates and analyzed under identical parameters. Identification was confirmed by matching MS/MS spectral similarity. Results refer to the Fig. S14. This targeted spatial metabolomics approach provides a robust framework for confident metabolite identification by combining the strengths of targeted acquisition (sensitivity, specificity) with orthogonal confirmation via standard comparison and MS/MS library matching. In general, if the matching score value is greater than 0.6, it is considered that the substance can be further qualitatively identified. The workflow is applicable to diverse tissue types and can be extended to isotope tracing and absolute quantification studies.

### Lipidomics

The long-chain fatty acid content of hVSMCs and mouse aortic VSMCs was determined using targeted lipidomics combined with GC-MS. To correct for lipid extraction efficiency and sample processing losses, a deuterated internal standard (nonadecanoic acid-d37, 10 μg/ml) was added immediately at the initiation of lipid extraction (prior to organic solvent addition). All procedures were performed on ice with rapid processing to minimize phospholipid degradation and prevent artificial release of PUFAs from storage lipids. Samples were gradually thawed at 4 °C, and aliquots were transferred to 5 ml of ice-cold dichloromethane-methanol (2:1, v/v), followed by vigorous vortexing. The mixture was washed with 2 ml of ice-cold Milli-Q water, and the lower organic layer was collected and dried under a gentle nitrogen stream. The residue was redissolved in 2 ml of *n*-hexane, and acid-catalyzed methylation was performed for 0.5 h to derivatize long-chain fatty acids into fatty acid methyl esters, which enables efficient ionization and accurate GC-MS detection. After adding 2 ml of Milli-Q water, 2 ml of the supernatant was collected, dried under nitrogen, redissolved in *n*-hexane, and transferred to an injection vial (injection volume, 1 μl; split ratio, 10:1). Chromatographic separation was performed using an Agilent 19091S-433UI capillary column (HP-5ms; 30 m × 250 μm × 0.25 μm). A quality control sample (pooled sample mix) was included in each analytical batch to monitor system stability and reproducibility (Fig. S15). MS was performed using an Agilent 5977B mass-selective detector (MSD) with the following parameters: injection port, 280 °C; ion source, 230 °C; transfer line, 250 °C; electron ionization energy, 70 eV; and SCAN mode/selected-ion-monitoring (SIM) mode detection. Peak areas and retention times were extracted using MSD ChemStation, and quantification was performed using a 7-point standard curve calibrated with the internal standard to account for matrix effects and extraction recovery.

### Primary cells isolation and culture

The aortas were carefully dissected from mice, followed by the manual removal of endothelial cells. The endothelium-denuded aortas from 5 mice were then pooled and cut into small pieces, each approximately 1 mm in dimension. To prepare a single-cell suspension, a mixed enzyme digestion solution was utilized containing collagenase I (450 U/ml), collagenase XI (250 U/ml), and hyaluronidase (120 U/ml; all from Sigma-Aldrich), along with deoxyribonuclease I (120 U/ml) obtained from Roche in Indianapolis, IN, USA. The digestion was carried out at 37 °C for a duration of 90 min. The resulting primary mouse VSMC single-cell suspension was then filtered through a 70-μm cell strainer. Subsequently, the cells were cultured in a 24-well plate using DMEM (Gibco) supplemented with 10% fetal bovine serum (Gibco) and 1% penicillin/streptomycin.

### Cell transfection

hVSMCs underwent transfection with plasmids overexpressing *ECH1* or *HuR* (Youbio) or with *ECH1*/*HuR*-specific small interfering RNAs (Table S9) using Lipofectamine 3000 (Invitrogen), following established protocols. Transfection duration was 48 h. The small interfering RNAs, synthesized by MiaoLingPlasmid (Hubei), were validated for knockdown efficiency via quantitative reverse transcription PCR and Western blotting, using β-actin as an internal control.

### RNA immunoprecipitation

RIP assays were conducted using a Millipore kit (Burlington) based on the manufacturer’s protocols. hVSMCs were lysed in 150 μl of RIP buffer, followed by 6-h incubation with 5 μg of anti-human immunoglobulin G (IgG) conjugated with magnetic beads or HuR antibodies (Abclonal). RNA–protein complexes were digested with proteinase K to isolate RNA, and HuR–*ECH1* mRNA interactions were quantified by qPCR.

### Statistical analysis

Data were analyzed using GraphPad Prism 10. Results are expressed as means ± SD, with *n* indicating mouse numbers or experimental replicates (see figure legends). When the data fit a normal distribution (normality was assessed using the Shapiro–Wilk test), 2-group comparisons used 2-tailed Student’s *t* tests, multigroup comparisons were conducted using analysis of variance (ANOVA), followed by post-hoc Tukey’s honestly significant difference tests. Statistical significance was defined as *P* < 0.05.

## Ethical Approval

All the experimental procedures were approved by the Animal Care Committee of the Army Medical University (AMUWEC20222973) and the Ethics Committee of Xinqiao Hospital (2024-Research063-02).

## Data Availability

Data will be made available from the corresponding author on reasonable request. Comprehensive experimental methods and supplementary information are presented in the expanded methods section and supplemental tables of the Supplementary Materials.

## References

[1] GBD 2019 Stroke Collaborators. Global, regional, and national burden of stroke and its risk factors, 1990-2019: A systematic analysis for the global burden of disease study 2019. Lancet Neurol. 2021;20(10):795–820. 34487721 10.1016/S1474-4422(21)00252-0 PMC8443449

[2] Vaduganathan M, Mensah GA, Turco JV, Fuster V, Roth GA. The global burden of cardiovascular diseases and risk: A compass for future health. J Am Coll Cardiol. 2022;80(25):2361–2371. 36368511 10.1016/j.jacc.2022.11.005

[3] Zhang S, Liu Y, Cao Y, Zhang S, Sun J, Wang Y, Song S, Zhang H. Targeting the microenvironment of vulnerable atherosclerotic plaques: An emerging diagnosis and therapy strategy for atherosclerosis. Adv Mater. 2022;34(29): Article e2110660. 35238081 10.1002/adma.202110660

[4] Covani M, Niccoli G, Fujimoto D, Scalamera R, Vergallo R, Porto I, McNulty I, Lee H, Kakuta T, Jang IK. Plaque vulnerability and cardiovascular risk factor burden in acute coronary syndrome: An optical coherence tomography analysis. J Am Coll Cardiol. 2025;86(2):77–89. 40634017 10.1016/j.jacc.2025.04.070

[5] Sun J, Singh P, Shami A, Kluza E, Pan M, Djordjevic D, Michaelsen NB, Kennbäck C, van der Wel NN, Orho-Melander M, et al. Spatial transcriptional mapping reveals site-specific pathways underlying human atherosclerotic plaque rupture. J Am Coll Cardiol. 2023;81(23):2213–2227. 37286250 10.1016/j.jacc.2023.04.008

[6] Bentzon JF, Otsuka F, Virmani R, Falk E. Mechanisms of plaque formation and rupture. Circ Res. 2014;114(12):1852–1866. 24902970 10.1161/CIRCRESAHA.114.302721

[7] Basatemur GL, Jørgensen HF, Clarke MCH, Bennett MR, Mallat Z. Vascular smooth muscle cells in atherosclerosis. Nat Rev Cardiol. 2019;16(12):727–744. 31243391 10.1038/s41569-019-0227-9

[8] Bennett MR, Sinha S, Owens GK. Vascular smooth muscle cells in atherosclerosis. Circ Res. 2016;118(4):692–702. 26892967 10.1161/CIRCRESAHA.115.306361 PMC4762053

[9] Grootaert MOJ, Bennett MR. Vascular smooth muscle cells in atherosclerosis: Time for a re-assessment. Cardiovasc Res. 2021;117(11):2326–2339. 33576407 10.1093/cvr/cvab046 PMC8479803

[10] Gaba P, Gersh BJ, Muller J, Narula J, Stone GW. Evolving concepts of the vulnerable atherosclerotic plaque and the vulnerable patient: Implications for patient care and future research. Nat Rev Cardiol. 2023;20(3):181–196. 36151312 10.1038/s41569-022-00769-8

[11] Bi X, Du C, Wang X, Wang XY, Han W, Wang Y, Qiao Y, Zhu Y, Ran L, Liu Y, et al. Mitochondrial damage-induced innate immune activation in vascular smooth muscle cells promotes chronic kidney disease-associated plaque vulnerability. Adv Sci. 2021;8(5): Article 2002738. 10.1002/advs.202002738 PMC7927614 33717842

[12] Ahn W, Burnett FN, Wojnar-Lason K, Doja J, Sreekumar A, Ghoshal P, Singla B, Gonsalvez G, Harris RA, Wang X, et al. Activation of receptor-independent fluid-phase pinocytosis promotes foamy monocyte formation in atherosclerotic mice. Redox Biol. 2024;78: Article 103423. 39615283 10.1016/j.redox.2024.103423 PMC11647241

[13] Wang Z, Chen S, Zhang F, Akhmedov S, Weng J, Xu S. Prioritization of lipid metabolism targets for the diagnosis and treatment of cardiovascular diseases. Research. 2025;8: Article 0618. 39975574 10.34133/research.0618 PMC11836198

[14] Jinson S, Zhang Z, Lancaster GI, Murphy AJ, Morgan PK. Iron, lipid peroxidation and ferroptosis play pathogenic roles in atherosclerosis. Cardiovasc Res. 2025;121(1):44–61. 39739567 10.1093/cvr/cvae270

[15] Liu YX, Yuan PZ, Wu JH, Hu B. Lipid accumulation and novel insight into vascular smooth muscle cells in atherosclerosis. J Mol Med. 2021;99(11):1511–1526. 34345929 10.1007/s00109-021-02109-8

[16] Mahmoudi M, Gorenne I, Mercer J, Figg N, Littlewood T, Bennett M. Statins use a novel Nijmegen breakage syndrome-1-dependent pathway to accelerate DNA repair in vascular smooth muscle cells. Circ Res. 2008;103(7):717–725. 18723444 10.1161/CIRCRESAHA.108.182899

[17] Cui Y, Chen Y, Li H, Zhang W, Wang X, Xia M, Gan N, Zhou Y, Li M, Zhang H, et al. PCSK9 promotes atherosclerotic plaque instability by inducing VSMC ferroptosis through the YAP1-NUPR1 axis. Research. 2025;8: Article 0922. 41064368 10.34133/research.0922 PMC12501616

[18] Ridker PM, Bhatt DL, Pradhan AD, Glynn RJ, MacFadyen JG, Nissen SE. Inflammation and cholesterol as predictors of cardiovascular events among patients receiving statin therapy: A collaborative analysis of three randomised trials. Lancet. 2023;401(10384):1293–1301. 36893777 10.1016/S0140-6736(23)00215-5

[19] Lee JY, Nam M, Son HY, Hyun K, Jang SY, Kim JW, Kim MW, Jung Y, Jang E, Yoon SJ, et al. Polyunsaturated fatty acid biosynthesis pathway determines ferroptosis sensitivity in gastric cancer. Proc Natl Acad Sci USA. 2020;117(51):32433–32442. 33288688 10.1073/pnas.2006828117 PMC7768719

[20] Wang R, Zhang J, Ren H, Qi S, Xie L, Xie H, Shang Z, Liu C. Dysregulated palmitic acid metabolism promotes the formation of renal calcium-oxalate stones through ferroptosis induced by polyunsaturated fatty acids/phosphatidic acid. Cell Mol Life Sci. 2024;81(1):85. 38345762 10.1007/s00018-024-05145-y PMC10861707

[21] Chao CC, Gutiérrez-Vázquez C, Rothhammer V, Mayo L, Wheeler MA, Tjon EC, Zandee SEJ, Blain M, de Lima KA, Takenaka MC, et al. Metabolic control of astrocyte pathogenic activity via cPLA2-MAVS. Cell. 2019;179(7):1483–1498.e1422. 31813625 10.1016/j.cell.2019.11.016 PMC6936326

[22] Thotala D, Craft JM, Ferraro DJ, Kotipatruni RP, Bhave SR, Jaboin JJ, Hallahan DE. Cytosolic phospholipase A2 inhibition with PLA-695 radiosensitizes tumors in lung cancer animal models. PLOS ONE. 2013;8(7): Article e69688. 23894523 10.1371/journal.pone.0069688 PMC3716600

[23] Zhou N, Yuan X, Du Q, Zhang Z, Shi X, Bao J, Ning Y, Peng L. FerrDb V2: Update of the manually curated database of ferroptosis regulators and ferroptosis-disease associations. Nucleic Acids Res. 2023;51(D1):D571–d582. 36305834 10.1093/nar/gkac935 PMC9825716

[24] Guryleva MV, Penzar DD, Chistyakov DV, Mironov AA, Favorov AV, Sergeeva MG. Investigation of the role of PUFA metabolism in breast cancer using a rank-based random Forest algorithm. Cancer. 2022;14(19): Article 4663. 10.3390/cancers14194663 PMC9562210 36230586

[25] Kagan VE, Mao G, Qu F, Angeli JP, Doll S, Croix CS, Dar HH, Liu B, Tyurin VA, Ritov VB, et al. Oxidized arachidonic and adrenic PEs navigate cells to ferroptosis. Nat Chem Biol. 2017;13(1):81–90. 27842066 10.1038/nchembio.2238 PMC5506843

[26] Li W, Deng X, Chen J. RNA-binding proteins in regulating mRNA stability and translation: Roles and mechanisms in cancer. Semin Cancer Biol. 2022;86(Pt 2):664–677. 35381329 10.1016/j.semcancer.2022.03.025 PMC9526761

[27] Zhou X, Zhao R, Lv M, Xu X, Liu W, Li X, Gao Y, Zhao Z, Zhang Z, Li Y, et al. ACSL4 promotes microglia-mediated neuroinflammation by regulating lipid metabolism and VGLL4 expression. Brain Behav Immun. 2023;109:331–343. 36791893 10.1016/j.bbi.2023.02.012

[28] Greco F, Bertagna G, Quercioli L, Pucci A, Rocchiccioli S, Ferrari M, Recchia FA, McDonnell LA. Lipids associated with atherosclerotic plaque instability revealed by mass spectrometry imaging of human carotid arteries. Atherosclerosis. 2024;397: Article 118555. 39159550 10.1016/j.atherosclerosis.2024.118555

[29] Ntshangase S, Khan S, Bezuidenhout L, Gazárková T, Kaczynski J, Sellers S, Rattray NJ, Newby DE, Hadoke PW, Andrew R. Spatial lipidomic profiles of atherosclerotic plaques: A mass spectrometry imaging study. Talanta. 2024;282: Article 126954. 39423636 10.1016/j.talanta.2024.126954

[30] Cao J, Martin-Lorenzo M, van Kuijk K, Wieland E, Gijbels MJ, Claes BSR, Heredero A, Aldamiz-Echevarria G, Heeren RMA, Goossens P, et al. Spatial metabolomics identifies LPC(18:0) and LPA(18:1) in advanced atheroma with translation to plasma for cardiovascular risk estimation. Arterioscler Thromb Vasc Biol. 2024;44(3):741–754. 38299357 10.1161/ATVBAHA.123.320278

[31] Morgan PK, Pernes G, Huynh K, Giles C, Paul S, Smith AAT, Mellett NA, Liang A, van Buuren-Milne T, Veiga CB, et al. A lipid atlas of human and mouse immune cells provides insights into ferroptosis susceptibility. Nat Cell Biol. 2024;26(4):645–659. 38589531 10.1038/s41556-024-01377-z

[32] Yang Y, Nawabi AQ, Yao Y, Liu N. Ferroptosis of smooth muscle cells in vascular diseases: From basic principles to clinical translation. Cell Death Discov. 2026;12(1):140. 41803100 10.1038/s41420-026-02950-1 PMC13040080

[33] Chen Z, Sun X, Li X, Liu N. Oleoylethanolamide alleviates hyperlipidaemia-mediated vascular calcification via attenuating mitochondrial DNA stress triggered autophagy-dependent ferroptosis by activating PPARα. Biochem Pharmacol. 2023;208: Article 115379. 36525991 10.1016/j.bcp.2022.115379

[34] Shi J, Wang QH, Wei X, Huo B, Ye JN, Yi X, Feng X, Fang ZM, Jiang DS, Ma MJ. Histone acetyltransferase P300 deficiency promotes ferroptosis of vascular smooth muscle cells by activating the HIF-1α/HMOX1 axis. Mol Med. 2023;29(1):91. 37415103 10.1186/s10020-023-00694-7 PMC10324182

[35] Wu H, Chen L, Lu K, Liu Y, Lu W, Jiang J, Weng C. HMGB2 deficiency mitigates abdominal aortic aneurysm by suppressing Ang-II-caused ferroptosis and inflammation via NF-κβ pathway. Mediat Inflamm. 2023;2023(1):2157355. 10.1155/2023/2157355 PMC10751175 38148870

[36] Li Y, Zhang L, Zhang Q, Zhang Y, Pan S, Zhao H, Zhang L. HSPB1 suppresses oxLDL-induced vascular smooth muscle cell ferroptosis by inhibiting DPP4. Arch Biochem Biophys. 2025;768: Article 110400. 40132776 10.1016/j.abb.2025.110400

[37] Chen Y, Cui Y, Li M, Xia M, Xiang Q, Mao Y, Li H, Chen J, Zeng W, Zheng X, et al. A novel mechanism of ferroptosis inhibition-enhanced atherosclerotic plaque stability: YAP1 suppresses vascular smooth muscle cell ferroptosis through GLS1. FASEB J. 2024;38(15): Article e23850. 39091212 10.1096/fj.202401251R

[38] Guo M, Xie L, Yuan H, Liao DF, Zheng XL. Catechin inhibits ox-LDL-induced ferroptosis in vascular smooth muscle cells to alleviate and stabilize atherosclerosis. Front Nutr. 2025;12:1594708. 40529424 10.3389/fnut.2025.1594708 PMC12171153

[39] Merino H, Parthasarathy S, Singla DK. Partial ligation-induced carotid artery occlusion induces leukocyte recruitment and lipid accumulation—A shear stress model of atherosclerosis. Mol Cell Biochem. 2013;372(1-2):267–273. 23054191 10.1007/s11010-012-1468-7

[40] Li C, Gao P, Zhuang F, Wang T, Wang Z, Wu G, Zhou Z, Xie H, Xie D, Zhao D, et al. Inhibition of ALOX12-12-HETE alleviates lung ischemia-reperfusion injury by reducing endothelial ferroptosis-mediated neutrophil extracellular trap formation. Research. 2024;7: Article 0473. 39268501 10.34133/research.0473 PMC11391482

[41] Liu M, Zheng J, Yu M, Wang Q, Yuan Y, Shao N, Yang X, Shen T, Wang L, Li A, et al. Stimuli-responsive CuFeTe_2_ nanosheets for amplified cuproptosis/ferroptosis in triple-negative breast cancer therapy. Adv Sci. 2025;13(3): Article e05739. 10.1002/advs.202505739 PMC12806277 41133938

[42] Ye Y, Chen A, Li L, Liang Q, Wang S, Dong Q, Fu M, Lan Z, Li Y, Liu X, et al. Repression of the antiporter SLC7A11/glutathione/glutathione peroxidase 4 axis drives ferroptosis of vascular smooth muscle cells to facilitate vascular calcification. Kidney Int. 2022;102(6):1259–1275. 36063875 10.1016/j.kint.2022.07.034

[43] Song W, Chen Y, Qin L, Xu X, Sun Y, Zhong M, Lu Y, Hu K, Wei L, Chen J. Oxidative stress drives vascular smooth muscle cell damage in acute Stanford type A aortic dissection through HIF-1α/HO-1 mediated ferroptosis. Heliyon. 2023;9(12): Article e22857. 38125409 10.1016/j.heliyon.2023.e22857 PMC10730757

[44] Lu L, Ye Y, Chen Y, Feng L, Huang J, Liang Q, Lan Z, Dong Q, Liu X, Li Y, et al. Oxidized phospholipid POVPC contributes to vascular calcification by triggering ferroptosis of vascular smooth muscle cells. FASEB J. 2024;38(7): Article e23592. 38581243 10.1096/fj.202302570R

[45] Liao M, Zou S, Wu J, Bai J, Liu Y, Zhi K, Qu L. METTL3-mediated m6A modification of NORAD inhibits the ferroptosis of vascular smooth muscle cells to attenuate the aortic dissection progression in an YTHDF2-dependent manner. Mol Cell Biochem. 2024;479(12):3471–3487. 38383916 10.1007/s11010-024-04930-4

[46] Wang N, Chen Z, Yao Y, Sun C, Wei W, Sun L, Zhang H, Li F, Butcher D, Sun SR, et al. Lipid metabolism drives dietary effects on T cell ferroptosis and immunity. Nature. 2026;653(8113):200–211. 41781622 10.1038/s41586-026-10193-4

[47] Yang WS, Kim KJ, Gaschler MM, Patel M, Shchepinov MS, Stockwell BR. Peroxidation of polyunsaturated fatty acids by lipoxygenases drives ferroptosis. Proc Natl Acad Sci USA. 2016;113(34):E4966–E4975. 27506793 10.1073/pnas.1603244113 PMC5003261

[48] Nassar ZD, Mah CY, Dehairs J, Burvenich IJ, Irani S, Centenera MM, Helm M, Shrestha RK, Moldovan M, Don AS, et al. Human DECR1 is an androgen-repressed survival factor that regulates PUFA oxidation to protect prostate tumor cells from ferroptosis. Elife. 2020;9: Article e54166. 32686647 10.7554/eLife.54166 PMC7386908

[49] Blomme A, Ford CA, Mui E, Patel R, Ntala C, Jamieson LE, Planque M, McGregor GH, Peixoto P, Hervouet E, et al. 2,4-dienoyl-CoA reductase regulates lipid homeostasis in treatment-resistant prostate cancer. Nat Commun. 2020;11(1):2508. 32427840 10.1038/s41467-020-16126-7 PMC7237503

[50] Keane DC, Takahashi HK, Dhayal S, Morgan NG, Curi R, Newsholme P. Arachidonic acid actions on functional integrity and attenuation of the negative effects of palmitic acid in a clonal pancreatic β-cell line. Clin Sci. 2011;120(5):195–206. 10.1042/CS20100282 PMC2990202 20840078

[51] Shoukry K, Schulz H. Significance of the reductase-dependent pathway for the beta-oxidation of unsaturated fatty acids with odd-numbered double bonds. Mitochondrial metabolism of 2-trans-5-cis-octadienoyl-CoA. J Biol Chem. 1998;273(12):6892–6899. 9506993 10.1074/jbc.273.12.6892

[52] Liu B, Yi W, Mao X, Yang L, Rao C. Enoyl coenzyme a hydratase 1 alleviates nonalcoholic steatohepatitis in mice by suppressing hepatic ferroptosis. Am J Physiol Endocrinol Metab. 2021;320(5):E925–e937. 33813878 10.1152/ajpendo.00614.2020

[53] Rao C, Qin H, Du Z. ECH 1 attenuates atherosclerosis by reducing macrophage infiltration and improving plaque stability through CD36 degradation. Arch Biochem Biophys. 2024;763: Article 110217. 39551333 10.1016/j.abb.2024.110217

[54] Finan JM, Sutton TL, Dixon DA, Brody JR. Targeting the RNA-binding protein HuR in cancer. Cancer Res. 2023;83(21):3507–3516. 37683260 10.1158/0008-5472.CAN-23-0972 PMC13449165

[55] Liu S, Jiang X, Lu H, Xing M, Qiao Y, Zhang C, Zhang W. HuR (human antigen R) regulates the contraction of vascular smooth muscle and maintains blood pressure. Arterioscler Thromb Vasc Biol. 2020;40(4):943–957. 32075416 10.1161/ATVBAHA.119.313897

[56] Wang YX, Zheng HY, Zhou K, Xie HL, Ren Z, Liu HT, Liu H, Zhou ZX, Jiang ZS. Multifaceted nature of HuR in atherosclerosis development. Curr Med Chem. 2025;32(17):3423–3437. 38310400 10.2174/0109298673279032231214110313

[57] Wang SF, Yang LY, Zhao AQ, Wang ZY, Wang S, Gong M, Zheng MQ, Liu G, Yang SY, Lin JJ, et al. A novel hidden protein p-414aa encoded by circSETD2(14,15) inhibits vascular remodeling. Circulation. 2025;151(22):1568–1582. 40099364 10.1161/CIRCULATIONAHA.124.070243

[58] Liu S, Jiang X, Cui X, Wang J, Liu S, Li H, Yang J, Zhang C, Zhang W. Smooth muscle-specific HuR knockout induces defective autophagy and atherosclerosis. Cell Death Dis. 2021;12(4):385. 33837179 10.1038/s41419-021-03671-2 PMC8035143

[59] Ma Z, Mao C, Chen X, Yang S, Qiu Z, Yu B, Jia Y, Wu C, Wang Y, Wang Y, et al. Peptide vaccine against ADAMTS-7 ameliorates atherosclerosis and postinjury neointima hyperplasia. Circulation. 2023;147(9):728–742. 36562301 10.1161/CIRCULATIONAHA.122.061516

[60] Kim SY, Moon TC, Chang HW, Son KH, Kang SS, Kim HP. Effects of tanshinone I isolated from salvia miltiorrhiza bunge on arachidonic acid metabolism and in vivo inflammatory responses. Phytother Res. 2002;16(7):616–620. 12410540 10.1002/ptr.941

[61] Huang R, Yong X, Li T, Wen H, Zhou X, Liao Y, You J, Yu C, Xu P, Wang Y, et al. 15-Lipoxygenase-2 deficiency induces foam cell formation that can be restored by salidroside through the inhibition of arachidonic acid effects. Open Life Sci. 2025;20(1):20251091. 40321157 10.1515/biol-2025-1091 PMC12048898

[62] Duvernay MT, Matafonov A, Lindsley CW, Hamm HE. Platelet Lipidomic profiling: Novel insight into cytosolic phospholipase A2α activity and its role in human platelet activation. Biochemistry. 2015;54(36):5578–5588. 26295742 10.1021/acs.biochem.5b00549 PMC7748375

[63] Liao P, Wang W, Wang W, Kryczek I, Li X, Bian Y, Sell A, Wei S, Grove S, Johnson JK, et al. CD8^+^ T cells and fatty acids orchestrate tumor ferroptosis and immunity via ACSL4. Cancer Cell. 2022;40(4):365–378.e366. 35216678 10.1016/j.ccell.2022.02.003 PMC9007863

[64] Tan H, Liu L, Qi Y, Zhang D, Zhi Y, Li Y, Zhang H, Liu J. Atorvastatin attenuates endothelial cell injury in atherosclerosis through inhibiting ACSL4-mediated Ferroptosis. Cardiovasc Ther. 2024;2024(1):5522013. 39742023 10.1155/2024/5522013 PMC11211010

[65] Hong Y, Feng J, Dou Z, Sun X, Hu Y, Chen Z, Liu L, Xu H, Du M, Tang P, et al. Berberine as a novel ACSL4 inhibitor to suppress endothelial ferroptosis and atherosclerosis. Biomed Pharmacother. 2024;177: Article 117081. 38971008 10.1016/j.biopha.2024.117081

[66] Yu H, Zhou C, Yang S, Yu J, Zhang X, Liang Z, Tan S, Song Y, Wang W, Sun Y, et al. Mitigation of arteriosclerosis through transcriptional regulation of ferroptosis and lipid metabolism by magnesium. Biomaterials. 2025;319: Article 123135. 39985976 10.1016/j.biomaterials.2025.123135

[67] Castillo AF, Orlando UD, Maloberti PM, Prada JG, Dattilo MA, Solano AR, Bigi MM, Ríos Medrano MA, Torres MT, Indo S, et al. New inhibitor targeting acyl-CoA synthetase 4 reduces breast and prostate tumor growth, therapeutic resistance and steroidogenesis. Cell Mole Life Sci. 2021;78(6):2893–2910. 10.1007/s00018-020-03679-5 PMC11072814 33068124

