# Supplementary material for: ECH1-Deficiency-Mediated Polyunsaturated Fatty Acid Accumulation Promotes Vascular Smooth Muscle Cell Ferroptosis and Chronic-Kidney-Disease-Associated Plaque Vulnerability
Source: Research (Wash D C). 2026 Sep 2;9:1411. doi: 10.34133/research.1411 (PMC13534806; doi:10.34133/research.1411)
Supplement: Supplementary file 1 [file research.1411.f1.docx]

**Supplementary Figures**

**Fig. S1**

**Fig. S1. PUFA accumulation is observed in fibrous cap VSMCs of CKD/ApoE^−/−^ mice.** (A) Violin plots of canonical cell-type-specific marker genes for cell annotation. (B-D) Quantification of the level of AA, LA and ALA in in mouse VSMCs treated with serum from CKD/ApoE-/- mice. Data represent mean ± SD. ****P* < 0.001, two-tailed Student’s t test.

**Fig. S2**

**Fig. S2.** **CKD induces VSMCs ferroptosis in vivo.** (A) Representative HE staining and quantification of the fraction area of multiple sections of aortic root plaques in Sham/*ApoE^-/-^* and CKD/*ApoE^-/-^* mice (n=10). Scale bar, 200 µm. (B) The time for carotid artery occlusion by thrombus in a FeCl3-induced thrombosis model in Sham/*ApoE^-/-^* and CKD/*ApoE^-/-^* mice (n=10). (C) Representative Masson staining and quantification of collagen of aortic root plaques in Sham/*ApoE^-/-^* and CKD/*ApoE^-/-^* mice (n=10). Scale bar, 100 µm. (D) Representative images and quantification of oil-red O staining in arteries of Sham/*ApoE^-/-^* and CKD/*ApoE^-/-^* mice. (E) Representative images of α-SMA IF staining in aortic root plaques of Sham/*ApoE^-/-^* and CKD/*ApoE^-/-^* mice. Scale bar, 10 µm. Quantification of the fold change of fibrous cap thickness and fibrous cap VSMC (α-SMA^+^) contents in aortic root plaques of Sham/*ApoE^-/-^* and CKD/*ApoE^-/-^* mice (n=10). (F and G) Relative mRNA expression levels of *GPX4* and *SLC7A11* in arteries of Sham/*ApoE*^-/-^ and CKD/*ApoE*^-/-^ mice (n=6). (H-J) WB analysis and quantification of GPX4 and SLC7A11 expression levels in arteries of Sham/*ApoE*^-/-^ and CKD/*ApoE*^-/-^ mice (n=6). Data represent mean ± (SD). ****P* < 0.001, two-tailed Student’s t test.

**Fig.S3**

**Fig. S3.** **CKD induces VSMCs ferroptosis in vitro.** (A) Representative TEM images of VSMCs in hVSMCs incubated with normal or CKD serum. Scale bar, 2 µm. (B and C) Representative images and quantification of relative MFI of DCFH-DA, Liperfluo and C11-BODIPY staining in hVSMCs incubated with normal or CKD serum. Scale bar, 50 µm (n=3). (D) Relative mRNA expression levels of *GPX4* and *SLC7A11* in hVSMCs after incubation with normal or CKD serum (n=3). (E) WB analysis and quantification of GPX4 and SLC7A11 expression levels in hVSMCs after incubation with normal or CKD serum (n=3). (I and J) Relative of MDA content and the ratio of GSH/GSSG in primary VSMCs after incubation with normal or CKD serum (n=5). (K and L) Representative FC analysis and the MFI of ROS and Liperfluo in primary VSMCs incubated with normal or CKD serum (n=3). Data represent mean ± SD, two-tailed Student’s t test. ****P*<0.001.

**Fig. S4**

**Fig. S4 Pharmacological inhibition of *cPLA2* mitigates VSMCs ferroptosis and plaque vulnerability.** (A-C) Quantification of the level of AA, LA and ALA in arteries of Sham/*ApoE^-/-^* and CKD/*ApoE^-/-^* mice after Giripladib treatment (n = 6). (D) The time for carotid artery occlusion by thrombus in a FeCl3-induced thrombosis model in Sham/*ApoE^-/-^* and CKD/*ApoE^-/-^* mice after Giripladib treatment (n=10). (E) Representative Masson staining and quantification of collagen of aortic root plaques in Sham/*ApoE^-/-^* and CKD/*ApoE^-/-^* mice after Giripladib treatment (n=10). Scale bar, 100 µm. (F-H) Quantification of the level of AA, LA and ALA in hVSMCs incubated with normal or CKD serum after Giripladib pretreatment (n = 6). (I and J) Relative of MDA content and the ratio of GSH/GSSG in hVSMCs incubated with normal or CKD serum after Giripladib pretreatment (n=3). (K) Representative TEM images of VSMCs in hVSMCs incubated with normal or CKD serum after Giripladib pretreatment. Scale bar, 2 µm. (L and M) Representative FC analysis and the MFI of ROS and Liperfluo in hVSMCs incubated with normal or CKD serum after Giripladib pretreatment (n=3). **P* < 0.05, ***P* < 0.01, ****P* < 0.001, ANOVA.

**Fig. S5**

**Fig. S5 *ECH1* overexpression attenuates CKD-Induced VSMC Ferroptosis.** (A) Representative images of ECH1 and α-SMA staining in human vascular samples from control and ESRD patients. Scale bar, 20 µm. (B and C) Representative mRNA quantification and WB analysis of *ECH1* in hVSMCs after *ECH1* plasmid transfection (n=3). (D) Relative mRNA expression levels of *GPX4* and *SLC7A11* in hVSMCs incubated with normal or CKD serum after *ECH1* overexpression (n=3). (E) WB analysis and quantification of GPX4 and SLC7A11 expression levels in hVSMCs incubated with normal or CKD serum after *ECH1* overexpression (n=3). (F-I) Representative images and quantification of relative MFI of DCFH-DA, Liperfluo and C11-BODIPY staining in hVSMCs incubated with normal or CKD serum after *ECH1* overexpression. Scale bar, 50 µm (n=3). (J and K) Representative FC analysis and the MFI of ROS and Liperfluo in hVSMCs incubated with normal or CKD serum after *ECH1* overexpression (n=3). ****P* < 0.001, ANOVA.

**Fig. S6**

**Fig. S6** **AA Supplementation Exacerbates CKD-Induced VSMC Ferroptosis. (A)** Quantification of the level of AA in hVSMCs incubated with normal or CKD serum after AA supplementation. (B-D) Representative images and quantification of relative MFI of DCFH-DA and Liperfluo staining in hVSMCs incubated with normal or CKD serum after AA supplementation. Scale bar, 50 µm (n=3). (E-H) Representative FC analysis and the MFI of ROS and Liperfluo in hVSMCs incubated with normal or CKD serum after AA supplementation (n=3). (I and J) Relative of MDA content and the ratio of GSH/GSSG in hVSMCs incubated with normal or CKD serum after AA supplementation (n= 3). Data represent mean ± SD. ****P* < 0.001, ANOVA.

**Fig. S7**

**Fig. S7 *ECH1* overexpression ameliorates AA-enhanced CKD-induced VSMC ferroptosis.** (A-C) Representative images and quantification of relative MFI of DCFH-DA and Liperfluo staining in hVSMCs incubated with normal or CKD serum after AA supplementation or *ECH1* overexpression. Scale bar, 50 µm (n=3). (D) Relative of MDA content and (E) the ratio of GSH/GSSG in hVSMCs incubated with normal or CKD serum after AA supplementation or *ECH1* overexpression (n=3). Data represent mean ± SD. ****P* < 0.001, ANOVA.

**Fig. S8**

**Fig. S8 HuR downregulation-induced *ECH1* deficiency in VSMCs under CKD milieus.** (A) Representative images of HUR and α-SMA staining in human vascular samples from control and ESRD patients. Scale bar, 20 µm. (B and C) WB analysis and mRNA quantification of *HuR* expression levels in hVSMCs after RNA interference of *HuR* (n=3). (D and E) WB analysis and mRNA quantification of *HuR* expression levels in hVSMCs after *HuR* overexpression (n=3). (F and G) WB analysis and detection of mRNA stability of *ECH1* expression levels in hVSMCs after RNA interference of *HuR* (n=3). Data represent mean ± SD. ****P* < 0.001, ANOVA.

**Fig. S9**

**Fig. S9 Genotypic Identification of *Ech1*^R26-LSL^-*ApoE^-/-^* and *Ech1*^Tagln-OE^-*ApoE^-/-^* Mice.** (A) Genetic identification of *Ech1* conditional knockin mice. (B and C) WB analysis and mRNA quantification of *ECH1* expression in arteries of *Ech1*^R26-LSL^-*ApoE^-/-^* and *Ech1*^Tagln-OE^-*ApoE^-/-^* mice (n=6). Data represent mean ± (SD). ****P* < 0.001, two-tailed Student’s t test.

**Fig. S10**

**Fig. S10 VSMC-specific overexpression of *ECH1* mitigates atherosclerotic plaque vulnerability.** (A and B) Representative Masson staining and quantification of collagen of aortic root plaques in Sham/*ApoE*^-/-^ and CKD/*ApoE*^-/-^ mice after *ECH1* overexpression. (n=6). Scale bar, 100 µm. (C) Quantification of necrotic area of aortic root plaques in Sham/*ApoE*^-/-^ and CKD/*ApoE*^-/-^ mice after *ECH1* overexpression. (n=6). (D) The time for carotid artery occlusion by thrombus in a FeCl3-induced thrombosis model in Sham/*ApoE^-/-^* and CKD/*ApoE^-/-^* mice after *ECH1* overexpression (n=6). Data represent mean ± SD. **P* < 0.05, ***P* < 0.01, ****P* < 0.001, ANOVA.

**Fig. S11**

**Fig. S11 Inhibition of *ACSL4* alleviates VSMCs ferroptosis. (A-C)** Quantification of the level of AA LA and ALA in hVSMCs incubated with normal or CKD serum after PRGL493 pretreatment. (D and E) Relative of MDA content and the ratio of GSH/GSSG in hVSMCs incubated with normal or CKD serum after PRGL493 pretreatment (n=3). (F) Representative TEM images of VSMCs in hVSMCs incubated with normal or CKD serum after PRGL493 pretreatment. Scale bar, 2 µm. (G-I) Representative images and quantification of relative MFI of DCFH-DA and Liperfluo staining in hVSMCs incubated with normal or CKD serum after PRGL493 pretreatment. Scale bar, 50 µm (n=3). (J and K) Representative FC analysis and the MFI of ROS and Liperfluo in hVSMCs incubated with normal or CKD serum after PRGL493 pretreatment (n=3). Data represent mean ± SD. ****P* < 0.001, ANOVA.

**Fig. S12**

**Fig. S12 Inhibition of *ACSL4* alleviates atherosclerotic plaque vulnerability of CKD/ApoE^−/−^ mice.** (A and B) Representative HE staining and quantification of the fraction area of multiple sections of aortic root plaques in Sham/*ApoE*^-/-^ and CKD/*ApoE*^-/-^ mice after PRGL493 treatment (n=10). Scale bar, 200 µm. (C) Representative Masson staining and quantification of collagen of aortic root plaques in Sham/*ApoE^-/-^* and CKD/*ApoE^-/-^* mice after PRGL493 treatment. Scale bar, 100 µm. (n=10). (D) The time for carotid artery occlusion by thrombus in a FeCl3-induced thrombosis model in Sham/*ApoE*^-/-^ and CKD/*ApoE*^-/-^ mice after PRGL493 treatment (n=10). Data represent mean ± SD. **P* < 0.05, ****P* < 0.01, ****P* < 0.001, ANOVA.

**Fig. S13**

**Fig. S13 Overexpression of *ECH1* combined with inhibition of *ACSL4* exerts a synergistic inhibitory effect on ferroptosis promotion in VSMCs induced by CKD.** (A-C) Representative images and quantification of relative MFI of DCFH-DA and Liperfluo staining in hVSMCs incubated with normal or CKD serum after ECH1 overexpression and PRGL493 pretreatment. (D and E) Relative of MDA content and the ratio of GSH/GSSG in hVSMCs incubated with normal or CKD serum after ECH1 overexpression and PRGL493 pretreatment. (n=3). Data represent mean ± SD. ***P* < 0.01, ****P* < 0.001, ANOVA.

**Fig.S14**

**Fig.S14. Peak extraction and qualitative analysis of the collected mass spectrometry data were conducted using the Bruker MetaboScape™ workstation. This figure displays the secondary mass spectrum information of AA, LA and ALA of the samples.**

**Fig. S15**

**Fig.S15.** **Quality control (QC) samples were utilized to evaluate the stability and reproducibility of the data, with a graph presenting the relative standard deviation (RSD) results of the analytes in the QC samples.**

**Fig. S16**

**Fig.S16. Graphic abstract.**
